# Review of Synthesis and Evaluation of Inhibitor Nanomaterials for Oilfield Mineral Scale Control

**DOI:** 10.3389/fchem.2020.576055

**Published:** 2020-11-19

**Authors:** Ping Zhang

**Affiliations:** Department of Civil and Environmental Engineering, Faculty of Science and Technology. University of Macau, Taipa, Macau

**Keywords:** oilfield, mineral, scale, inhibitor, nanomaterial, transport, porous medium

## Abstract

Oilfield flow assurance is the subject to study the impact on the flow of production fluids due to physicochemical changes in the production system. Mineral scale deposition is among the top 3 water-related flow assurance challenges in petroleum industry, particularly for offshore and shale operations. Scale deposition can lead to serious operational risks and significant financial loss. The most commonly adopted strategy in oilfield scale control is the deployment of chemical inhibitors. Although conventional chemical inhibitors are effective in inhibiting scale threat, they have the drawbacks of short transport distance and limited squeeze lifetime due to their intrinsic chemical properties. In the past decade, as an alternative to conventional chemical inhibition, research efforts have been made to prepare functional nanomaterials with different chemical compositions to overcome the drawbacks of conventional chemical inhibitors. These synthesized nanomaterials can serve as delivery vehicles to deploy inhibitors into the target location in the production system. These nanomaterials are reported to have multiple advantages over the conventional inhibitors in terms of transportability, controlled release, and functionality, evidenced by a series of experimental studies. This review presents an overview of scale inhibitor nanomaterial development and the current methods to synthesize and to evaluate these nanomaterials in a systematic and comprehensive manner. This review focuses on the chemistry principles and methodologies underlying inhibitor nanomaterial synthesis and also the chemical instrument and strategies in evaluating the physiochemical properties of these materials in terms of inhibition effectiveness, transportability, and inhibitor return. The scale inhibitor nanomaterials (SINMs) presented in this review exemplify the continuous development in our capabilities in adopting novel nanotechnology in combating actual engineering challenges in petroleum industry.

## Introduction

The subject of flow assurance is a relatively new domain of engineering and research in the petroleum industry. Flow assurance is the study of hydrocarbon flow from the reservoir to the point of scale in an economical and safe fashion (Gudmundsson, 2017). Decades ago when hydrocarbons were primarily produced from conventional onshore fields, flow-assurance-related operational issues were less pronounced. However, with the significant developments in offshore deepwater and shale fields in the past two decades, the associated flow assurance challenges greatly threaten oilfield operational integrity and safety (Mackay et al., 2005; Vazquez et al., 2016; Vankeuren et al., 2017; Zhang et al., 2017a; Chang et al., 2019). Common oilfield flow assurance threats include gas hydrate blockage, mineral scale formation, asphaltene deposition, pipe corrosion, and wax (paraffin) formation (Gao et al., 2006; Kelland, 2006, 2011, 2014; Frenier and Ziauddin, 2008; Zerpa et al., 2011; Wang et al., 2018; Qasim et al., 2019). Together with pipe corrosion and gas hydrate blockage, mineral scale deposition is one of the top 3 produced water-related flow assurance threats (Frenier and Ziauddin, 2008; Kelland, 2014; Vazirian et al., 2016; Zhang et al., 2019a). Mineral scale (hereafter referred to as “scale”) is the hard inorganic crystal deposit from the water phase (Cowan and Weintritt, 1976). In the past few decades, extensive research efforts have been made in both academia and industry to control scale threats, especially for oilfield operations (Frenier and Ziauddin, 2008; Kelland, 2014; Hu and Mackay, 2017; Ribeiro et al., 2017; Ishkov et al., 2019). The primary strategy for scale control replies on chemical inhibition using scale inhibitor chemicals (Vazquez et al., 2016, 2019). It has been confirmed in laboratory experiments and field observations that chemical inhibitors can effectively delay mineral scale formation and control scale threat. However, due to the intrinsic chemical properties of these inhibitor products, they have a short transport distance in reservoir formation, indicative of a limited scale protection area in formation (Zhang et al., 2010, 2011a; Stamatiou and Sorbie, 2020). Furthermore, chemical inhibitors have a relatively short squeeze lifetime during squeeze treatment with a significant amount of inhibitors being removed from the formation prematurely (Tomson et al., 2006, 2008). As a consequence, the idea of formulating scale inhibitor nanomaterials (SINMs) has been proposed as an alternative strategy for oilfield scale control (Shen et al., 2008; Zhang et al., 2017b). With appropriate surface chemistry modifications, these nanomaterials are able to transport through formation porous medium and considerably extend the squeeze lifetime. It should be noted that the definition of nanomaterials differs in the literature in terms of size dimension restriction of either 100 or 1,000 nm (Letchford and Burt, 2007).

**Scheme 1:**
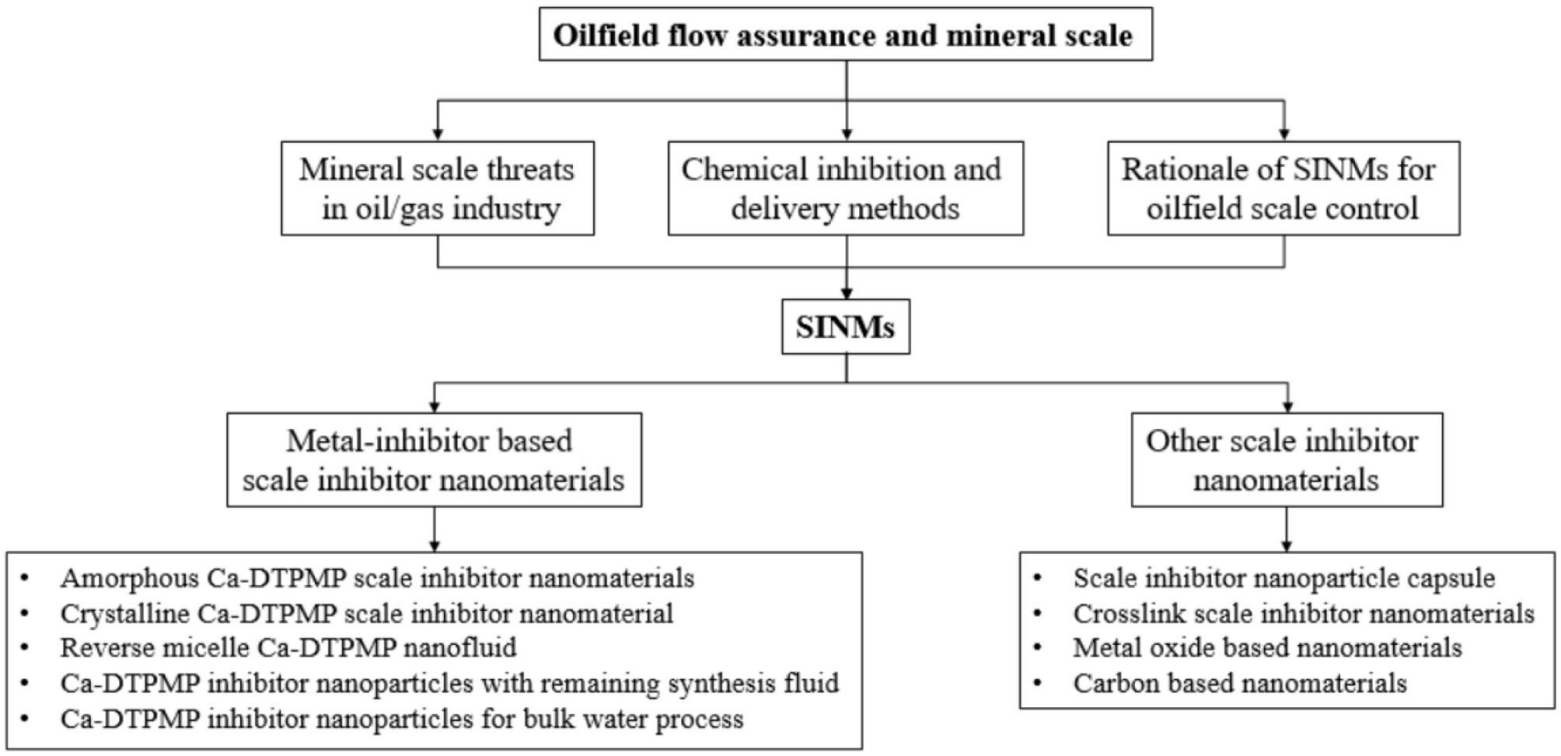
Schematic of this review article.

Recently, active researches have been carried out in applying nanotechnology in the oil and gas industry. The objective of these studies is to make nanotechnology a greater contribution to oilfield technological advances. Some of the existing study areas include enhanced oil recovery, stimulation, drilling and completion, refining and processing, etc. (Kong and Ohadi, 2010; Kelland, 2014; Yang et al., 2015; Bera and Belhaj, 2016; Liu H. et al., 2016). For instance, Olayiwola and Dejam (2019) carried out a comprehensive review of the interaction between nanomaterials and low salinity water and surfactant for the goal of enhanced oil recovery in sandstone and carbonate fields. In the domain of flow assurance, laboratory investigations have been conducted to inhibit asphaltene deposition using metal oxide nanoparticles (Mohammadi et al., 2011). In another study, nanoparticles have been adopted for production tubing dewaxing (Haindade et al., 2012). As elaborated above, a key potential advantage of the nanoparticles relative to the conventional chemicals is their superior migration capacity in formation porous medium. Extensive publications can be found on the topic of the transport of different classes of nanoparticles in reservoir formation (Wu et al., 2012; Kini et al., 2014; Zhang T. et al., 2015). Although a number of existing review articles cover the topic of scale inhibition using nanomaterials, these reviews are generally sporadic and non-systematic. This review presents an overview of scale inhibitor nanomaterial development and the methods to synthesize and to evaluate these nanomaterials in a systematic and comprehensive manner. This review focuses on the chemistry principles and methodologies underlying SINM synthesis and also the chemical instrument and strategies in evaluating the physiochemical properties of SINM in terms of inhibition effectiveness, transport in formation medium, and inhibitor return. The objective of this review is to keep the readers abreast of the development in the field of oilfield scale inhibitor nanomaterial development. The schematic of this review article is shown in Scheme 1.

## Oilfield Flow Assurance and Mineral Scale

### Mineral Scale Threats in Oil/Gas Industry

The formation of scale particles is a result of exceeding local saturation of the brine solution at the operational conditions of concern (Jordan et al., 2008a; Kan and Tomson, 2012; Zhang P. et al., 2015; Dai et al., 2019; Kan et al., 2019; Ness and Sorbie, 2019; Zhang et al., 2019b). The solution supersaturation will initiate scale crystal formation and subsequently promote the crystal growth of scale particles. The formed scale particles will eventually precipitate out of water phase and deposit onto the surface of surrounding environments (Mackay, 2003; Webster et al., 2014; Zhang P. et al., 2018). Scale deposition can occur anywhere in the petroleum production system from subsurface to processing facilities (Fink, 2011). Scale particles can substantially reduce the throughput of the production tubing or flow line, leading to a considerable reduction in fluid flowrate. At extreme cases, the conduit is completely blocked by the deposited scale particles. Scale formation can also form in the formation subsurface (Cosultchi et al., 2002; Hajirezaie et al., 2019). During water injection campaigns, seawater can be injected into the formation to enhance oil recovery and to maintain reservoir pressure. The commingling of seawater and formation water can result in sulfate scale formation in the reservoir pore space, reducing formation porosity, and damaging formation integrity (Frenier and Ziauddin, 2008). Other than blockage-related production threats, scale particles can deposit on the surface of various processing facilities, impairing the effectiveness of these equipment (Chaussemier et al., 2015; Li et al., 2015; Touati et al., 2018). With the substantial developments in offshore and shale fields in the past decade, the harsh operating conditions exacerbate scale threats. In offshore productions, the increasing water depth is associated with higher operation temperature and pressure. Coupled with the elevated salinity, scaling threats can take place in subsurface, production tubing, subsea flowlines, and topsides facilities (Fan et al., 2012a). It has been reported that scale deposition is responsible for ~20% of downtime for a major oil company across all regions (Vazquez et al., 2007). In shale play, water evaporation can be enormous due to hydraulic fracturing and pressure drawdown, resulting in a severe scaling threat (Vankeuren et al., 2017; Chang et al., 2019). The most commonly observed mineral scales are carbonates and sulfates. Carbonates, such as calcium carbonate (calcite), are generally formed due to the reduction in system pressure, which causes CO_2_ gas to evolve from water phase into gas phase and subsequently an increase in solution pH (Chen et al., 2005; Shi et al., 2013; Kelland, 2014; Chaussemier et al., 2015). Sulfates, such as barium sulfate (barite), are typically water injection associated in the subsurface formation (Fan et al., 2010, 2011; Yan C. et al., 2015; Carvalho et al., 2017; Yan et al., 2017).

### Chemical Inhibition and Delivery Methods

**Figure 1:**
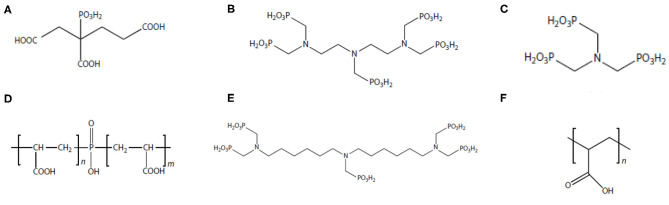
Molecular structure of common oilfield scale inhibitors. **(A)** 2-Phosphono butane-1,2,4-tricarboxylic acid (PBTCA); **(B)** diethylenetriaminepentakis (methylenephosphonic acid) (DTPMP); **(C)** aminotris(methylenephosphonic acid) (ATMP); **(D)** polyphosphinocarboxylic acid (PPCA); **(E)** bis-hexamethylenetriamine-penta(methylene phosphonic acid) (BHPMP); **(F)** polyacrylic acid (PAA).

In oilfield operations, the most commonly adopted approach in controlling scale threat is to apply chemical scale inhibitors (Zhang P. et al., 2015; Bukuaghangin et al., 2016; Liu Y. et al., 2016). Scale inhibitors are a class of specialty chemicals, which can kinetically delay mineral scale formation and subsequent precipitation (Wang et al., 2013, 2014; Yan F. et al., 2015; Zhang et al., 2019c). The molecular structures of several common oilfield scale inhibitors are shown in Figure 1. Although mineral scale deposition will eventually occur in spite of the presence of inhibitor, kinetically delaying scale formation and deposition is of vital importance in managing scale threat in oilfield operations (Setta and Neville, 2011; Fan et al., 2012b; Farooqui and Sorbie, 2016; Sanni et al., 2017). This is because it is substantially difficult and extraordinarily more expensive to remediate scale damage in downhole formation or bottom of the well-compared to further upstream places such as processing facilities. It has been reported that the cost to remediate scale damage in a subsea well can be as high as tens of millions USD (Zhang et al., 2017a). In addition, the operational risks associated with downhole scale remediation and removal are considerably more than that at processing facilities (Cosultchi et al., 2002; Hajirezaie et al., 2019). Thus, oilfield strategy in scaling threat control, especially in subsurface and subsea production systems, is to inhibit or delay scale formation via chemical inhibition. Scale inhibitors fall into the category of threshold inhibitors since scale inhibitors are able to control scale threat at a very low concentration of a few milligrams per liter or lower (Kan and Tomson, 2012; Kelland, 2014; Kan et al., 2019). The mechanisms of scale inhibitor in inhibiting scale include crystal growth modification and nucleation prevention (Kan and Tomson, 2012; Kelland, 2014; Kan et al., 2019). Two most commonly used oilfield scale inhibitors are aminophosphonates (phosphonates) and polymers (Shaw et al., 2012a,b). Laboratory testing shows that phosphonates are more efficient than most of the polymeric inhibitors. However, the drawback of the phosphonates is that these compounds contain phosphorus element, which can pollute the environment. Majority of the scale inhibitors are water soluble. Thus, the inhibitor product needs to be dissolved in the water phase of the production fluid to inhibit scale formation.

**Figure 2:**
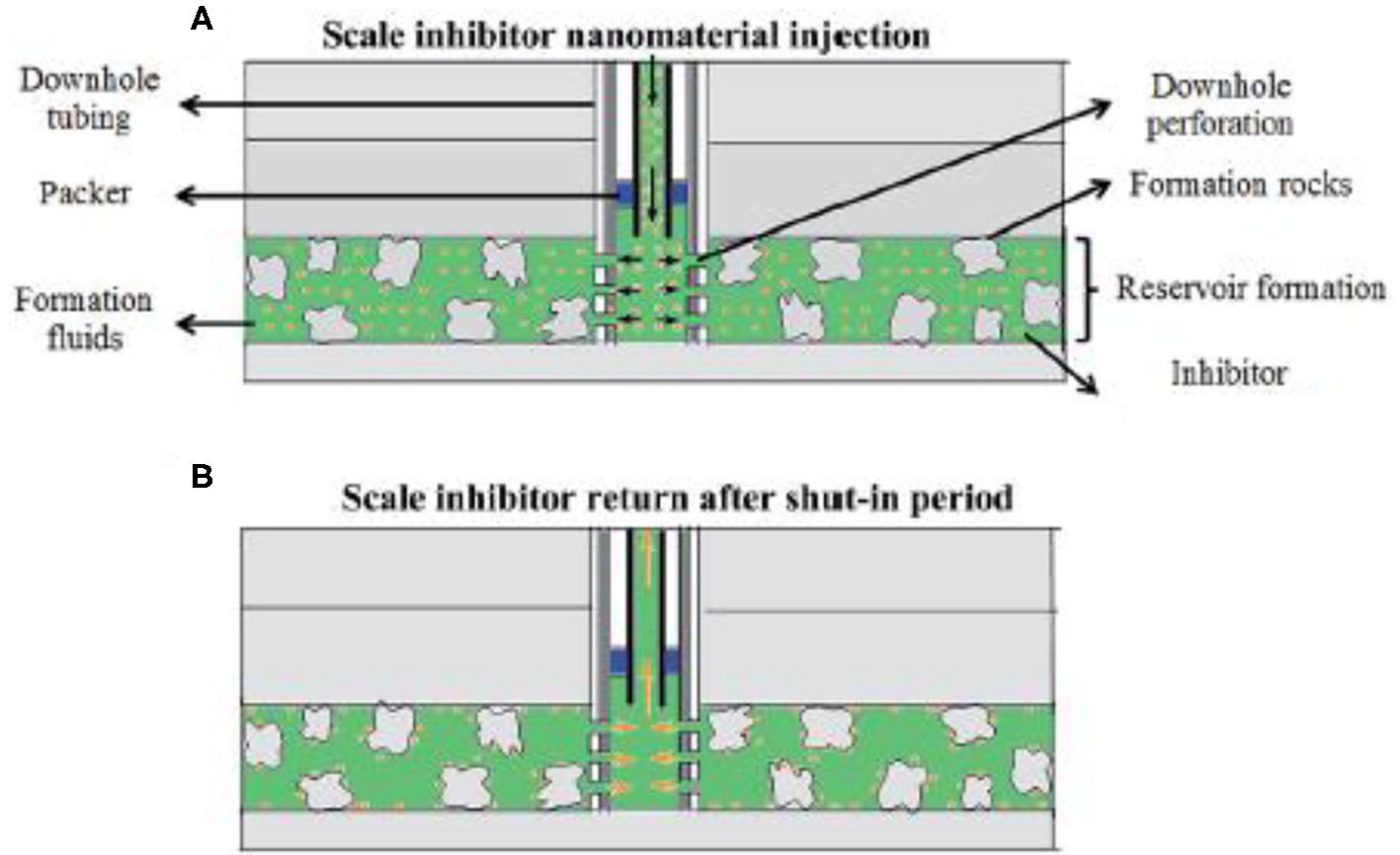
Schematic of the field scale squeeze operation. **(A)** The process of injecting nanomaterial into formation; **(B)** the process of returning inhibitor after the shut-in period. Reproduced from Zhang et al. (2016a) with permission from The Royal Society of Chemistry.

A key issue associated with oilfield scale control is the delivery of scale inhibitor to the target zone. As for scale control at locations of wellhead and other upstream locations, scale inhibitors are delivered via continuous injection (Frenier and Ziauddin, 2008; Fink, 2011; Zhang P. et al., 2018; Zhang Z. et al., 2018; Zhang et al., 2019d). An injection skid will be mounted at the wellhead so that scale inhibitor can be injected continuously. The injected inhibitor will commingle with the production fluid by dissolving into the produced water for scale control. The injection rate can be adjusted according to the production rate to ensure that the aqueous phase inhibitor concentration exceeds the minimum inhibitor concentration (MIC). By dissolving into the produced water, scale inhibitor will flow with the production fluid to upstream production locations to control scaling threat. However, inhibitor delivery to bottom of well or downhole formation is much more complicated. To achieve this goal, a scale squeeze treatment is normally conducted by injecting (“squeezing”) a volume of scale inhibitors through the production tubing into wellbore formation (Frenier and Ziauddin, 2008), as depicted in Figure 2.

During a typical squeeze treatment, the production well will be shut down followed by a preflush treatment where a volume of brine fluid is injected into the wellbore formation for the objective of cleaning up reservoir matrix free from deposits and oil. Subsequently, a volume of scale inhibitor solution (also called pill solution) is injected (squeezed) into the wellbore formation, followed by another volume of overflush fluid to push the injected inhibitors into deeper formation. Next, the well will continue to be shut in for a period of several hours to allow the injected inhibitor to attach to the surface of the formation materials. Afterwards, the well will be put back online, and the production fluids will flow from reservoir into the production well (Fink, 2011). In this way, the production fluid, mainly the produced water, will flow over the formation material surface, and the retained inhibitors will be released into the produced water for scale control in wellbore and bottom of well. The concentration of the released inhibitor in the produced water (also called return concentration) will be closely monitored. Once the return concentration drop below MIC, another squeeze treatment campaign will be required for downhole scale control. The time duration from the completion of the squeeze treatment to the time point where return concentration is below MIC is called the squeeze lifetime. Obviously, it is of great economic and operational significance to extend the squeeze lifetime (Vazquez et al., 2013, 2014). With regard to the retention mechanism of the inhibitors, some scholars suggest that the inhibitors are surface adsorbed by formation materials, and the presence of divalent metals in formation facilitates the surface adsorption via complexation (Sorbie et al., 1997; Vazquez et al., 2011; Jarrahian and Sorbie, 2020). As a consequence, the release process of the inhibitor into the production fluid during the resumed production is controlled by the desorption of the inhibitor from the formation surface (Vazquez et al., 2011). Another school of thoughts believes that the inhibitors are retained by forming solid precipitate with divalent metals in wellbore formation (Tomson et al., 2006, 2008). The inhibitor pill solution is normally acidic, and thus, the acid in the pill can dissolve formation matrix, releasing divalent metals, such as Ca^2+^ or Mg^2+^ species (Kan et al., 2004). Subsequently, the injected inhibitors will precipitate with these metal ions forming metal-inhibitor precipitates on the surface of formation materials. During the resumed production, these precipitates will gradually dissolve into the produced water, releasing inhibitor chemicals for scaling control (Shaw and Sorbie, 2014).

### Rationale of SINMs for Oilfield Scale Control

As elaborated above, scale squeeze treatment is an important engineering solution in oilfield to control downhole and wellbore scaling threat. The drawback of the conventional scale squeeze treatment is that due to the adsorption or precipitation reaction in the wellbore formation, the chemical inhibitors have a limited migration capacity in the formation, leading to a relatively small protection area against scale damage near the wellbore. The longer the distance the inhibitor chemicals can transport to, the larger the protection area near the wellbore. Furthermore, it is commonly observed that approximately one-third of the injected inhibitors will be rapidly flushed out of the formation reservoir shortly after the onset of resumed production. This is mainly caused by the fact that a large fraction of the injected inhibitor chemicals is freely mobile in the formation pore space, instead of being retained by the formation materials. Because of this phenomenon, the squeeze lifetime is greatly shortened compared to otherwise. Thus, it will be desirable to come up with an engineering approach capable of placing the inhibitor farther away from the wellbore and extending the squeeze life. With these considerations, the idea of preparing SINM has been conceived to address these operational needs in oilfield scale treatment. In conventional squeeze treatment, the injected inhibitors will inevitably interact with the divalent metal species, forming metal-inhibitor complex/precipitate. Thus, one can conceive that it is possible to prepare a metal-inhibitor solid material with a regulated particle size and a designated surface chemistry at laboratory setting. One requirement of the prepared solid material is that this material needs to be stable in brine solution at representative oilfield conditions in order to be delivered into deeper formation. This can be achieved by particle size control and particle surface chemistry modification. In addition, this material needs to be able to release scale inhibitor chemical in a controlled manner. In other words, this prepared material should possess the capacity of gradually releasing inhibitors into the produced water at representative oilfield conditions with a return concentration above MIC for a prolonged duration. Furthermore, it will be desirable to be able to regulate the return concentration in order to handle scale threats of different severity levels. Since nanomaterials have a small particle size with a strong enough surface potential with tunable surface properties and morphologies, the prepared metal-inhibitor solid materials normally have a particle size of several hundreds of nanometers and thus are termed SINMs. Other than metal-inhibitor materials, other nanomaterials are also reported in the literature for the objective of scaling control, and these materials will be reviewed in the below section as well.

## Metal-Inhibitor-Based Scale Inhibitor Nanomaterials

### Amorphous Ca-DTPMP Scale Inhibitor Nanomaterials

**Figure 3:**
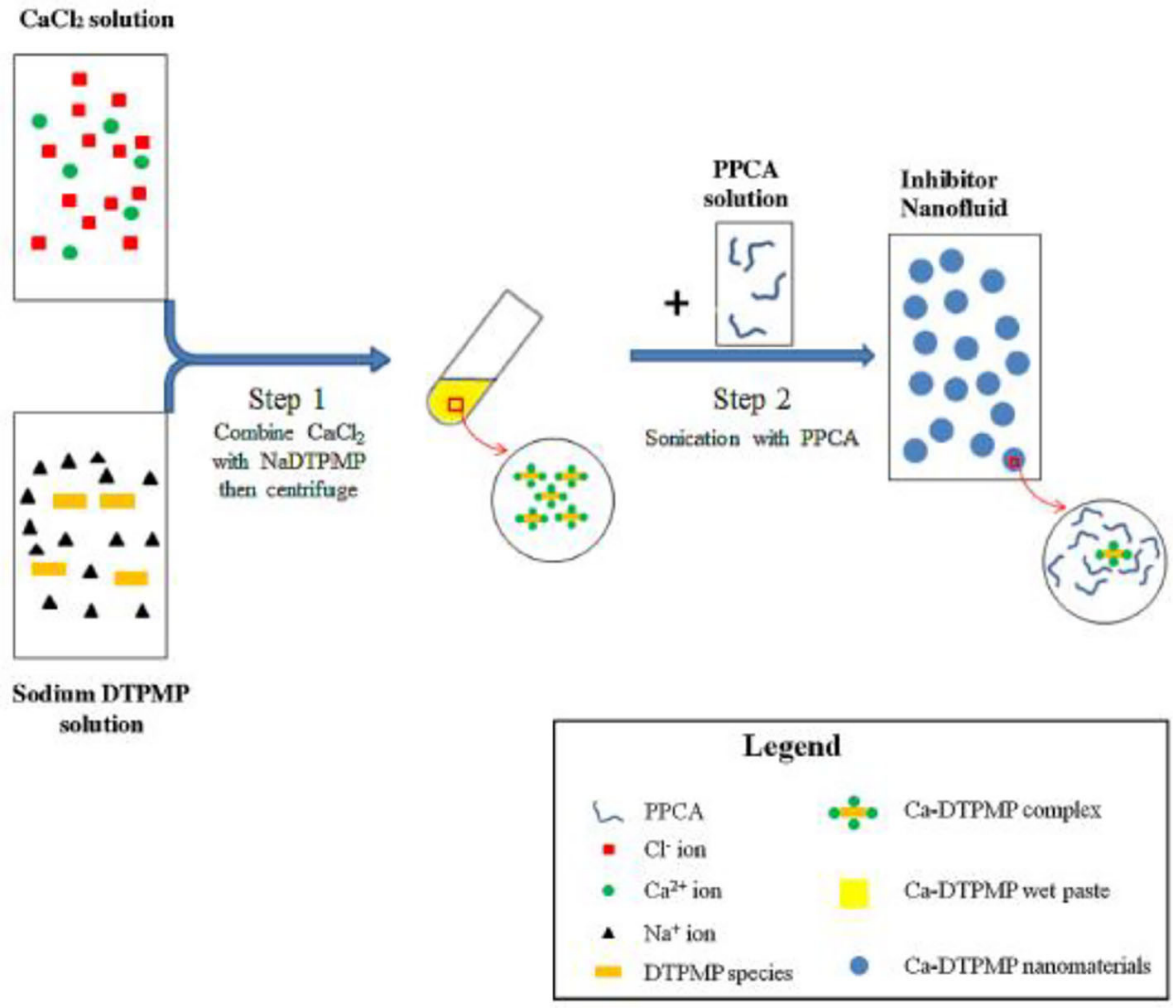
Schematic diagram of the synthesis procedure of PPCA modified Ca-DTPMP nanofluid. Reprinted from Zhang et al. (2017b). Copyright (2017) with permission from Elsevier.

**Figure 4:**
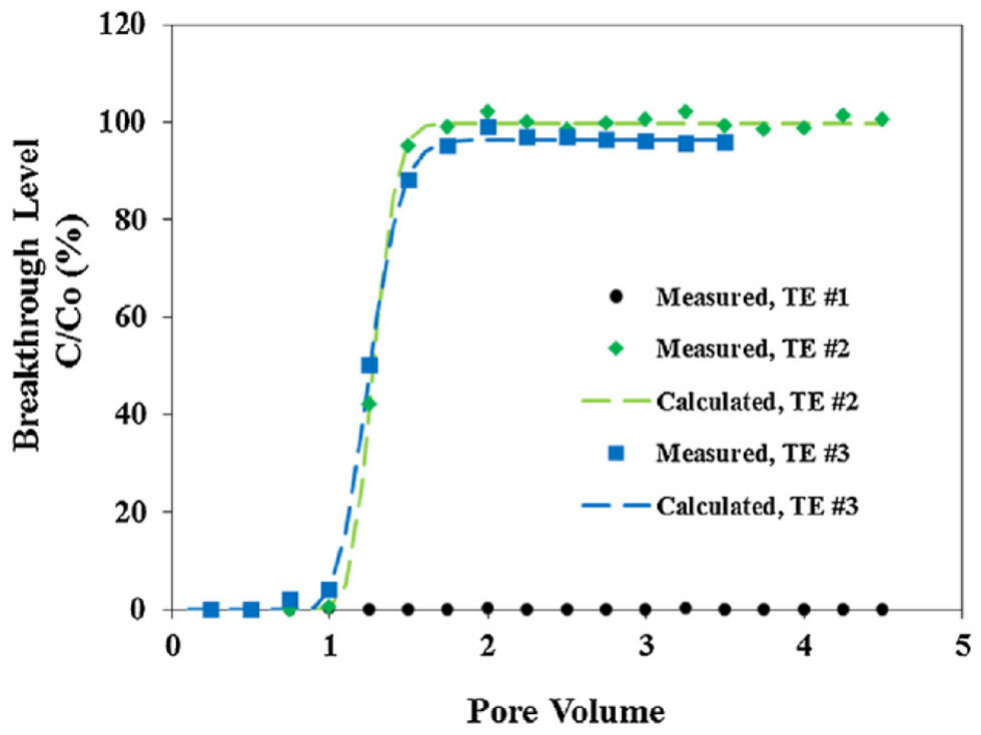
Scale inhibitor nanomaterial breakthrough curves in calcite medium. The x-axis is the pore volume of the nanofluid, and y-axis is the breakthrough level (C/C_o_). The dot, diamond, and square markers represent the experimentally measured breakthrough levels from three transport experiments: TE #1 with no KCl or PPCA; TE #2 with no KCl but 0.2% PPCA; and TE #3 with 0.2% KCl and 0.2% PPCA. The dashed lines denote the calculated breakthrough levels based on the mathematical solution to Equation (1). Reprinted from Zhang et al. (2017b). Copyright (2017) with permission from Elsevier.

Perhaps the first metal-inhibitor SINM was reported by Shen et al. (2008) in a Society of Petroleum Engineers (SPE) conference proceeding, which was later published as a journal publication (Zhang et al., 2017b). In this study, a common oilfield phosphonate inhibitor diethylenetriamine pentakis (methylene phosphonic acid) (DTPMP) was mixed with Ca^2+^ ion to form Ca-DTPMP precipitate. The formed Ca-DTPMP solid was further developed into SINM via centrifugation treatment in the presence of a polymer phosphino-polycarboxylic acid (PPCA), as depicted in Figure 3. PPCA is a polymeric scale inhibitor in its own rights. The presence of PPCA not only reduces the SINM particle size but also increases the zeta potential of the particle. In the presence of PPCA, the size of the prepared Ca-DTPMP SINM was determined to be between 100 and 200 nm, and the zeta potential is −30 mV, suggesting of a desirable aqueous stability. X-ray diffraction (XRD) characterization suggests that this material is amorphous. Similar to this pivotal study, metal-phosphonate SINMs were also prepared by surfactant-assisted synthesis approach (Zhang et al., 2010) and silica-templated method (Zhang et al., 2011a). Following the SINM synthesis, a key question to be resolved is the transportability of SINM in formation media. In order to evaluate this, an experimental column setup has been established to examine the migration capacity of SINM in formation porous medium at representative oilfield conditions. It shows that the presence of PPCA can considerably prompt the transport of such materials in calcite porous medium compared with virtually zero transportability in the absence of PPCA, as illustrated in Figure 4.

**Figure 5:**
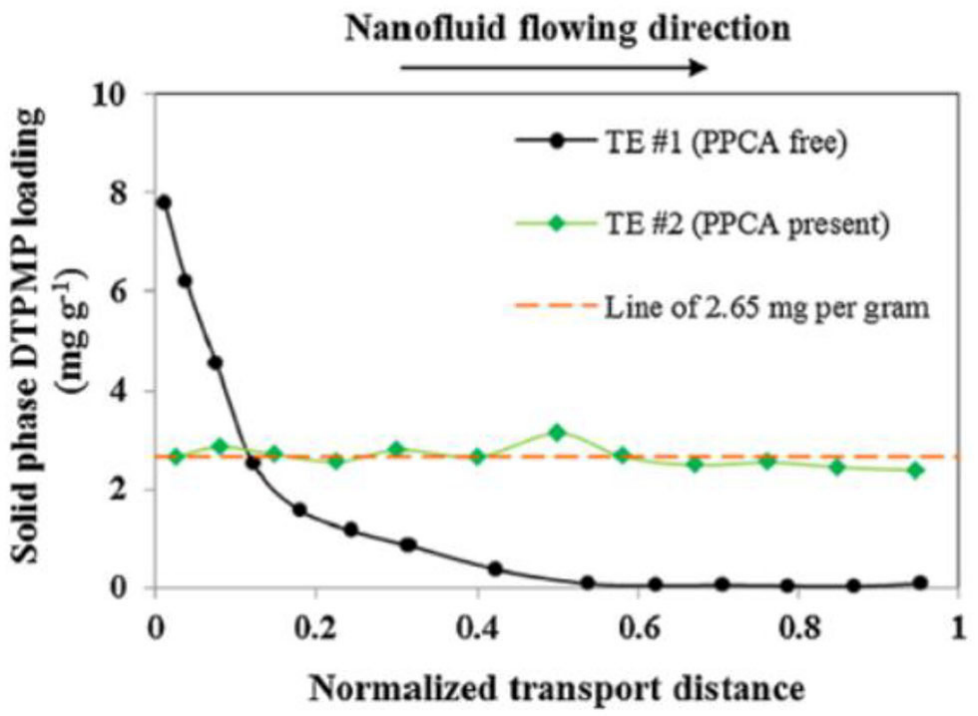
DTPMP solid phase distribution in calcite medium after TE #1 (black dots) and TE #2 (green diamonds). The red dashed line represents 2.65 mg g^−1^, which is the average DTPMP content calculated for TE #2. Reprinted from Zhang et al. (2017b). Copyright (2017) with permission from Elsevier.

Calcite (calcium carbonate) material was selected as the column packing material since calcite is the most active component in reacting with phosphonate inhibitors (Zhang et al., 2010, 2016b). Furthermore, the DTPMP solid phase distribution in the calcite medium was investigated after the completion of the transport study. It shows that the PPCA surface-modified Ca-DTPMP SINM is relatively uniformly distributed across the entire length of the packed column, as shown in Figure 5. The role of PPCA on Ca-DTPMP SINM was summarized by the authors as (1) PPCA modifies the particle surface morphology and increases the zeta potential and (2) reverses the surface change to allow an electrostatic repulsive force to exist between SINM and calcite particles.

The transport experimental data can be fitted via a one-dimensional advection dispersive equation (1-D ADE) (Charbeneau, 2006; Clark, 2009):

(1) $$\begin{array}{l} R\frac{\partial C}{\partial t}=D\frac{\partial^{2}C}{\partial x^{2}}-v\frac{\partial C}{\partial x}-J_{\text{d}}C \end{array}$$

where *C* (mg L^−1^) denotes the effluent SINM concentration, *t* (min) is the time, *x* (cm) represents the transport distance inside the column, *D* (cm^2^ min^−1^) corresponds to the dispersion coefficient, and *v* (cm min^−1^) is the linear pore velocity. *R* represents the retardation factor, and *J*_d_ (min^−1^) is the SINM deposition rate coefficient to medium surfaces. *J*_d_ value can be calculated based upon the filtration theory (Ryan and Elimelech, 1996; de Jonge et al., 2004):

(2) $$J_{\text{d}}=-\frac{v}{L}ln\left(\frac{c^{\prime }}{C_{0}^{\;\prime }}\right)$$

where *L* (cm) corresponds to the length of the column. *C*′/*C*′_0_ denotes the final breakthrough level. In another study, the transport of SINM in calcite medium was evaluated from the viewpoint of the particle filtration and attachment theory (Zhang et al., 2016a). SINM transport can be regarded as the process of particle collection and attachment. Thus, one can adopt collection efficiency (η_0_) and attachment efficiency (α) to elaborate the SINM collection and attachment to the surface of formation medium (Clark, 2009).

(3) $$\alpha =-\frac{2d_{c}}{3\left(1-\varepsilon \right)L\eta_{0}}\text{ln}\left(\frac{c^{\prime }}{C_{0}^{\;\prime }}\right)$$

where *d*_c_ is the diameter of the medium, and ε is the medium porosity. The increase in η_0_ and α value suggests of an increase in the tendency for SINM particle removal by the surface of medium. Equation (3) can be rearranged to calculate the transport distance of SINM in porous medium. By assuming that the maximum transport distance occurs when the breakthrough level (*C*′/*C*′_0_) is 1%, SINM maximum transport distance (*L*_MAX_) can be calculated as:

(4) $$\begin{array}{l} L_{MAX}=-\frac{2d_{c}}{3\left(1-\varepsilon \right)\alpha \eta_{0}}\text{ln}(0.01) \end{array}$$

**Figure 6:**
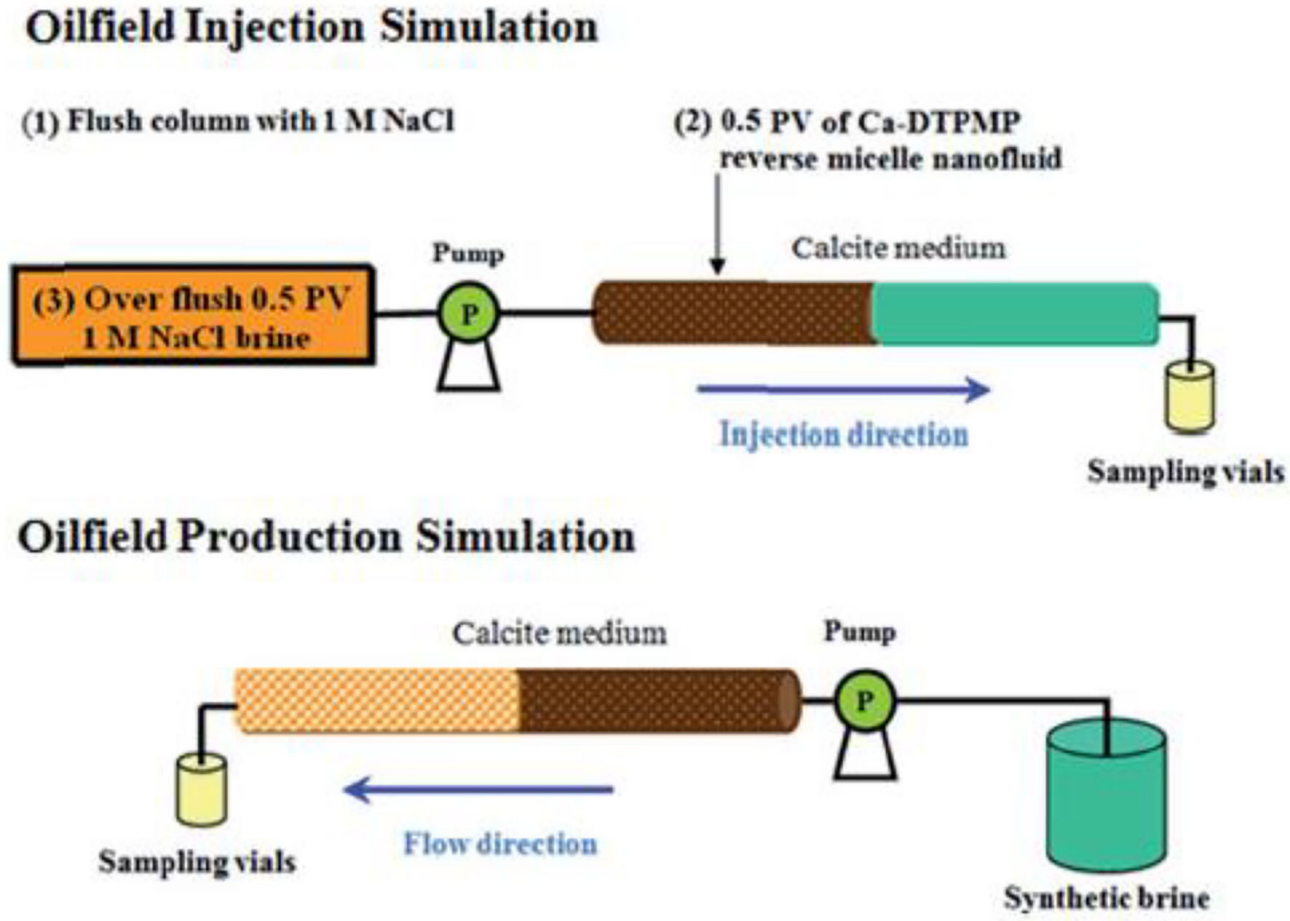
Schematic diagram of laboratory squeeze simulation test including the two stages of injection simulation and production simulation. Reproduced from Zhang et al. (2016e) with permission from The Royal Society of Chemistry.

**Figure 7:**
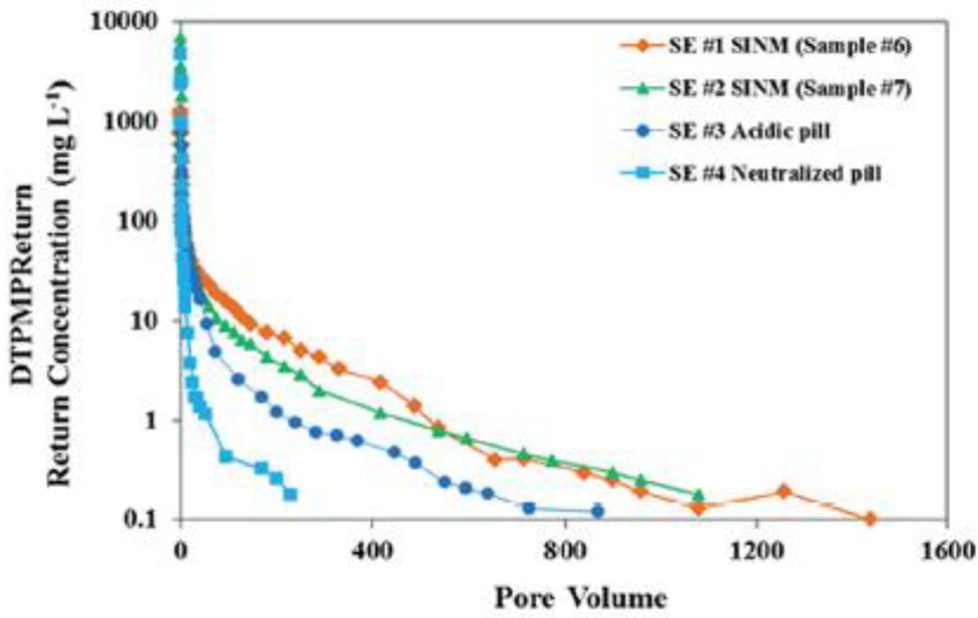
Laboratory squeeze simulation results using scale inhibitor nanomaterials in sandstone medium. Four DTPMP return profiles were included: SE #1 using SINM with 2% KCl; SE #2 using SINM with 1% KCl; SE #3 using a conventional acidic pill; SE #4 using a conventional neutralized pill. Reproduced from Zhang et al. (2016a) with permission from The Royal Society of Chemistry.

Another critical aspect of SINM properties is the ability to release inhibitor into the produced water for scale control. Ideally, SINM can release inhibitor at a concentration level above MIC in a controlled manner for an extended period of time. The inhibitor return of the prepared Ca-DTPMP SINM was studied by laboratory squeeze simulation test, illustrated in Figure 6. The squeeze simulation test includes the injection and production simulation stages to mimic the actual field squeeze treatment. As shown in Figure 7, compared with the conventional pill (both acidic and neutralized pills), PPCA-modified Ca-DTPMP SINM demonstrated an extended squeeze lifetime, which is mainly driven by the fact that the initial inhibitor return concentration from SINM is much lower than that from the conventional pills. As discussed by the authors, the reason for the reduced initial return in SINM is because there is limited freely mobile inhibitor in the formation pore space relative to the conventional pills. Thus, a much reduced amount of inhibitor was flushed out of the column within the first 10 pore volume (PV) of return flow at representative oilfield conditions. PV is characterized as the column empty space for the fluid to occupy. One PV in laboratory investigation can be viewed as equivalent to one pore volume of formation in the field (Wheaton, 2016). It is worthwhile to mention that the concentrations of phosphonate and phosphonate-based SINMs are normally measured based upon the aqueous concentration of phosphorus element. Measurement of phosphonate concentration can be achieved using inductively coupled plasma. As for low concentration of phosphonate (lower than a few milligrams per liter), phosphonate was first digested forming phosphate, followed by the formation of phosphomolybdenum blue complex, which is measured spectrophotometrically (Kan et al., 1994).

**Figure 8:**
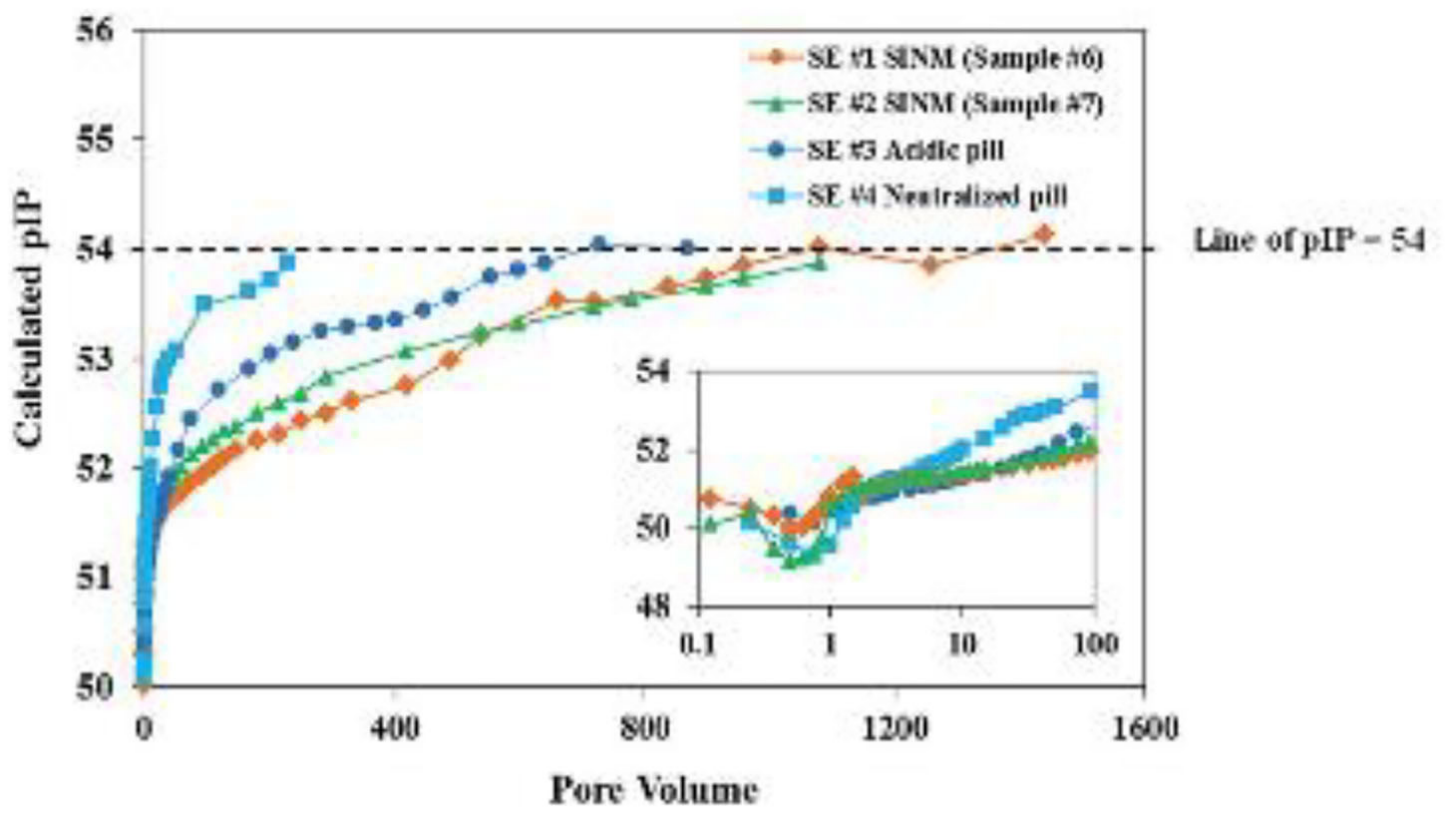
The calculated negative logarithm of ion activity product (pIP) of the four return profiles detailed in Figure 6 captions. The smaller inserted figure is part (up to 100 PV) of the main figure. The dashed line represents pIP of 54, which is close to the calculated final pIP. Reproduced from Zhang et al. (2016a) with permission from The Royal Society of Chemistry.

In order to obtain a deeper insight on Ca-DTPMP SINM return process, the aqueous phase DTPMP return concentration was evaluated from the perspectives of acid–base chemistry and Ca–ligand complexation (Zhang et al., 2016a). The negative logarithm of the Ca-DTPMP ion activity product (pIP) was calculated by assuming a molecular formula of Ca_3_H_4_DTPMP following the below equation:

(5) $$\begin{array}{l} \text{pIP}=-{\text{log}}_{10}[{\left({\text{Ca}}^{2+}\right)}^{3}{\left\{{\text{H}}^{+}\right\}}^{4}({\text{DTPMP}}^{10-})] \end{array}$$

where braces correspond to activity of species and parentheses to molar concentration. A previously developed speciation model has been adopted to calculated the free Ca^2+^ (Ca^2+^) and free DTPMP^10−^ (DTPMP^10−^) concentrations (Tomson et al., 1994). Computational results on the pIP values throughout the SINM return duration suggest that the calculated pIP increases from 50 to 54, indicating a considerable reduction in SINM aqueous solubility over several orders of magnitude, as shown in Figure 8. These authors argued that the reduction in pIP values are indicative of a phase transition phenomenon where the initial form of SINM was amorphous and during the course of inhibitor return; this material was gradually developed into a crystalline solid, which accounts for the much reduced return concentration in the later section of inhibitor return. With this observation, efforts have been made to prepare crystalline Ca-DTPMP SINM in a laboratory setting as reviewed below.

### Crystalline Ca-DTPMP Scale Inhibitor Nanomaterial

**Figure 9:**
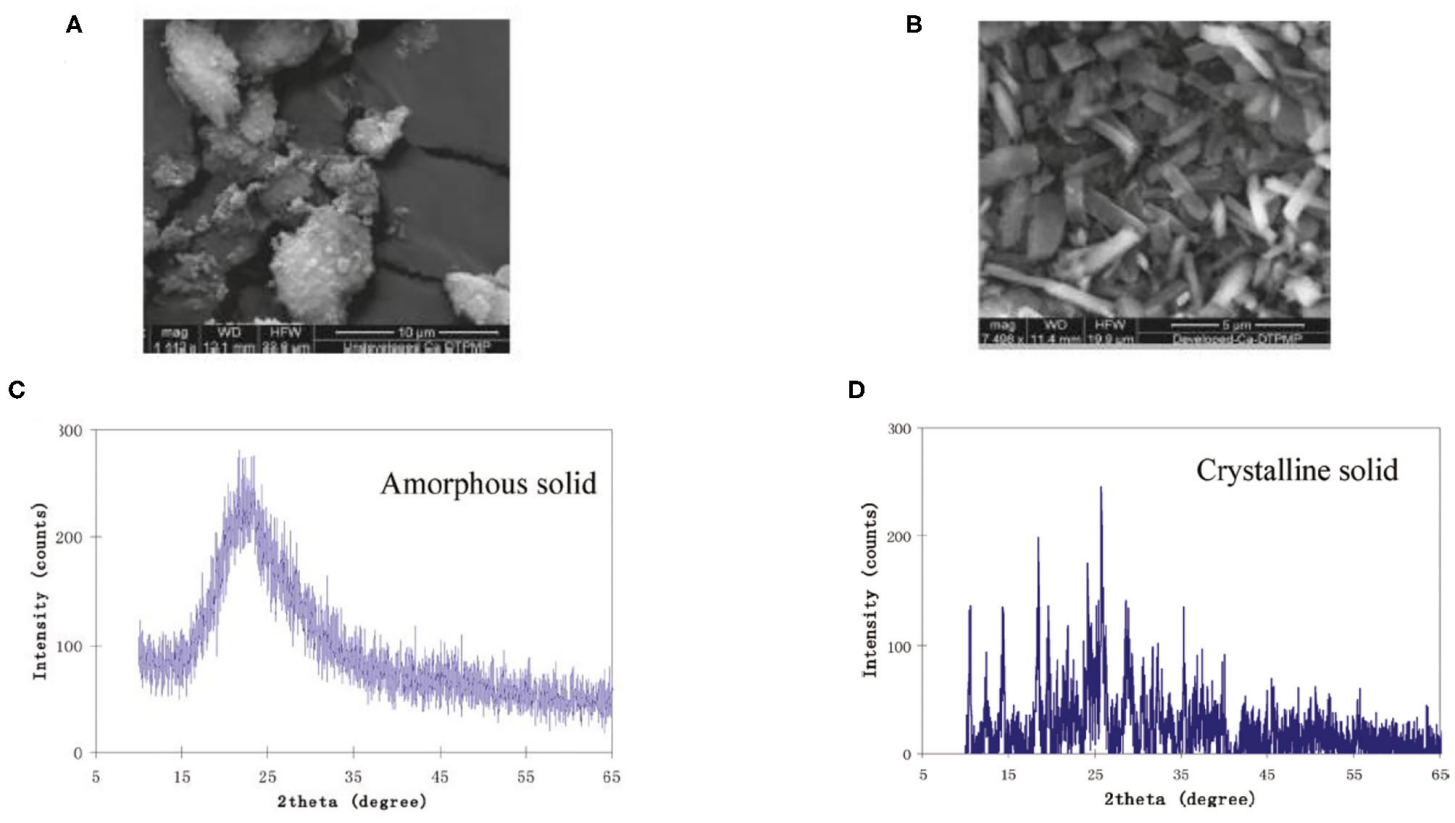
SEM characterization of **(A)** amorphous phase Ca-DTPMP before diafiltration treatment and **(B)** crystalline Ca-DTPMP solid after diafiltration. XRD characterization of **(C)** amorphous phase Ca-DTPMP before diafiltration treatment and **(D)** crystalline Ca-DTPMP solid after diafiltration. Reprinted with permission from Zhang et al. (2011b). Copyright (2011) American Chemical Society.

Kan et al. (1994) published the very first study to elaborate the existence of a crystalline phase Ca-DTPMP material, which has an aqueous solubility of a few orders of magnitude lower than its amorphous counterpart. This observation was made at a representative oilfield condition with a wide range of calcium and DTPMP concentrations. In another study, both static and dynamic dissolution tests suggest that the Ca-DTPMP solid phase transition from amorphous to crystalline is a continuous process with a middle phase of an amorphous/microcrystalline structure being observed by XRD and scanning electron microscopy (SEM) characterizations (Zhang et al., 2016b). Based on the aforementioned laboratory studies on Ca-DTPMP synthesis, experimental studies have been carried out to prepare crystalline Ca-DTPMP SINM with a low solubility. The advantage of such a material is that due to its low solubility, this material should be able to return DTPMP inhibitor at a much lower return concentration, which means that the squeeze lifetime will be greatly extended for a given amount of inhibitor squeezed into formation. The first reported synthesis study of crystalline Ca-DTPMP was conducted using silica nanomaterials as the template (Zhang et al., 2011b). The synthesis approach involves mixing of a DTPMP containing solution with another Ca–silica slurry solution to form a white precipitate of Ca-DTPMP. Silica serves as the template for SINM synthesis due to its low point of charge and high aqueous stability. Following the formation of Ca-DTPMP slurry, this material was further developed into a crystalline phase by adopting a diafiltration cell setup. By combining dialysis and filtration, the diafiltration cell allows a continuous flow of a Ca-containing brine through the formed Ca-DTPMP solid to realize phase transition. In addition, sodium dodecylbenzene sulfonate (SDBS) surfactant was adopted in the synthesis study to modify the surface chemistry of the SINM. As reported by these authors, SDBS can effectively reduce zeta potential of SINM, leading to an enhanced aqueous stability. XRD and SEM characterizations of the prepared SINM confirmed the crystallinity of this material, as illustrated in Figure 9. Transport experiments suggest that the crystalline material is able to transport through formation porous medium, and the breakthrough level was substantially impacted by the pore velocity. In addition, during SINM transport through porous medium, SINM removal by the medium is controlled by a diffusion mechanism. Thus, it is proposed that altering the injection strategy of SINM can manage the migration of these materials in formation medium. In another study, the transportability of SINM was further investigated by evaluating the impact of surfactant preflush on SINM migration and placement in the formation media of calcite and sandstone (Zhang et al., 2016c). Experimental results show that surfactant preflush treatment can considerably enhance the transport of silica-based Ca-DTPMP SINM in the tested formation media. This observation was driven by the substantial increase in the interaction energy between SINM and medium surfaces, which is calculated by the Derjaguin–Landau–Verwey–Overbeek (DLVO) theory. The calculation of the interaction energy involves the calculation of electric double-layer repulsion energy and London–van der Waals attraction energy.

**Figure 10:**
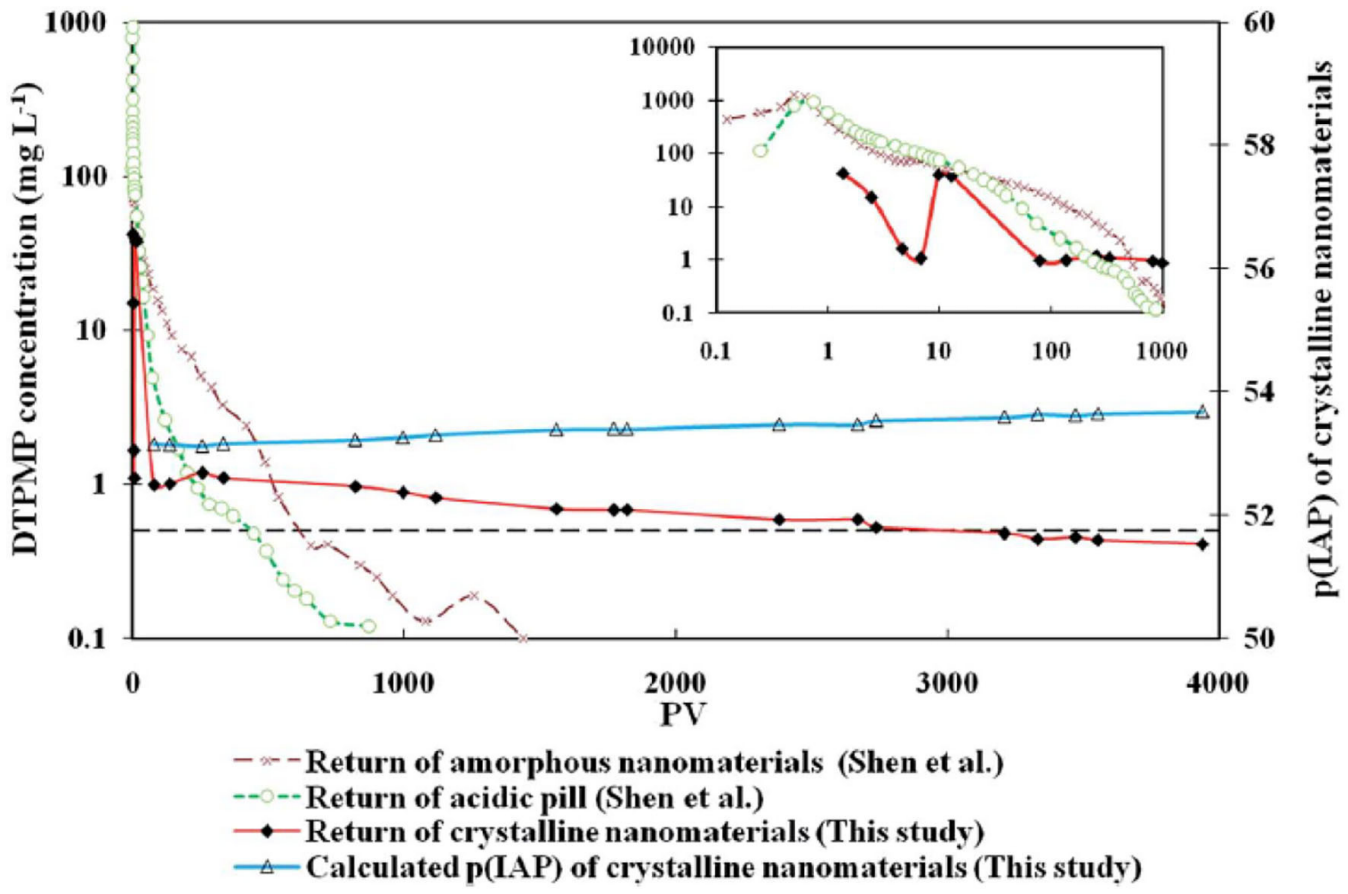
The long-term flow back performance of the crystalline Ca-DTPMP SINM in squeeze simulation test in sandstone medium. Three returns curves were from a crystalline SINM, an acidic pill solution, and an amorphous nanomaterial fluid. The insert shows the DTPMP return concentrations within the first 1,000 PVs. The dashed line represents 0.5 mg L^−1^ of DTPMP. Reproduced from Zhang et al. (2016c) with permission from The Royal Society of Chemistry.

The long-term inhibitor return of the prepared crystalline Ca-DTPMP SINM was studied by laboratory squeeze simulation tests. It shows that the crystalline SINM is able to return DTPMP stably at a return concentration level of 0.5–1 mg L^−1^ for almost 4,000 PVs. On the contrary, acidic DTPMP pill and amorphous Ca-DTPMP SINM can only maintain a return duration of several 100 PVs and around 1,200 PVs, respectively. As shown in the return profile (Figure 10), the return DTPMP concentrations for both acidic pill and amorphous SINM reached over 1,000 mg L^−1^ within the first few PVs, indicating that a significant amount of DTPMP was released from the formation media shortly after the onset of inhibitor return. Crystalline SINM, on the other hand, shows a considerably reduced return concentration of around 50 mg L^−1^ after around 1 PV return, and the return concentration quickly reduced to 1 mg L^−1^ at 100 PVs and stayed relatively stable at this level until 1,000 PVs. The reduced inhibitor return concentration in the very first stage of crystalline SINM return is attributed to the low aqueous solubility of this material. Based upon the calculated pIP values following Equation (5), crystalline SINM has a pIP value of 53.7, where this value was around 50 for amorphous SINM or conventional pill. Due to the greatly reduced solubility of crystalline SINM relative to conventional pill and amorphous SINM, there is a limited amount of crystalline SINM being released into the brine water via dissolution. Consequently, the crystalline SINM can gradually release DTPMP inhibitor to the brine during the squeeze simulation test, leading up to an extended squeeze lifetime. As shown in both Figures 7, **10**, toward the end of squeeze simulation tests by adopting SINMs, the aqueous concentration of the inhibitor becomes as low as 0.2 and 0.5 mg L^−1^, respectively. The effectiveness of scale inhibitors at a given concentration can be estimated using ScaleSoftPitzer™ software (Version 2020) (Kan et al., 2019). It shows that an aqueous concentration of BHPMP inhibitor of 0.2 and 0.53 mg L^−1^ can effectively inhibit barite scale for over 2 h with an SI (barite) of 1.44 and 1.63, respectively, at 27 atm and 60°C with 1 M NaCl ionic strength condition.

Following the previous study on crystalline SINM synthesis, a more recent study introduced a facile one-pot synthesis method to prepare low-solubility Ca-DTPMP material (Ruan et al., 2020). In this method, citrate was used to form complex with Ca^2+^. The Ca–citrate complex is subsequently mixed with DTPMP solution to produce Ca-DTPMP precipitate. The complexation of citrate with Ca^2+^ can considerably reduce the free Ca^2+^ concentration in the aqueous phase and gradually release Ca^2+^ into the aqueous phase to form Ca-DTPMP precipitate during the synthesis process. Due to the substantial reduction in free Ca^2+^ in the aqueous phase, the formed Ca-DTPMP, without additional phase transition development, shows a low solubility in aqueous phase, leading to a prolonged squeeze lifetime in the squeeze simulation test. Compared to the earlier studies where Ca-DTPMP phase transition involved diafiltration setup or column apparatus (Zhang et al., 2011b, 2016b), this study suggests that a low-solubility Ca-DTPMP material can be prepared via a one-pot synthesis route in a facile and economical manner. Furthermore, in this study, efforts have been made to prepare non-Ca-based inhibitor materials, including Mg/Zr-DTPMP and Mg/Ca-DTPMP. These inhibitor materials are able to return DTPMP inhibitor with an extended squeeze lifetime with different inhibitor return concentrations. This suggests that the stabilized return concentration from these solid inhibitor materials can be adjusted by modifying the composition of the metal-inhibitor nanomaterials.

### Reverse Micelle Ca-DTPMP Nanofluid

**Figure 11:**
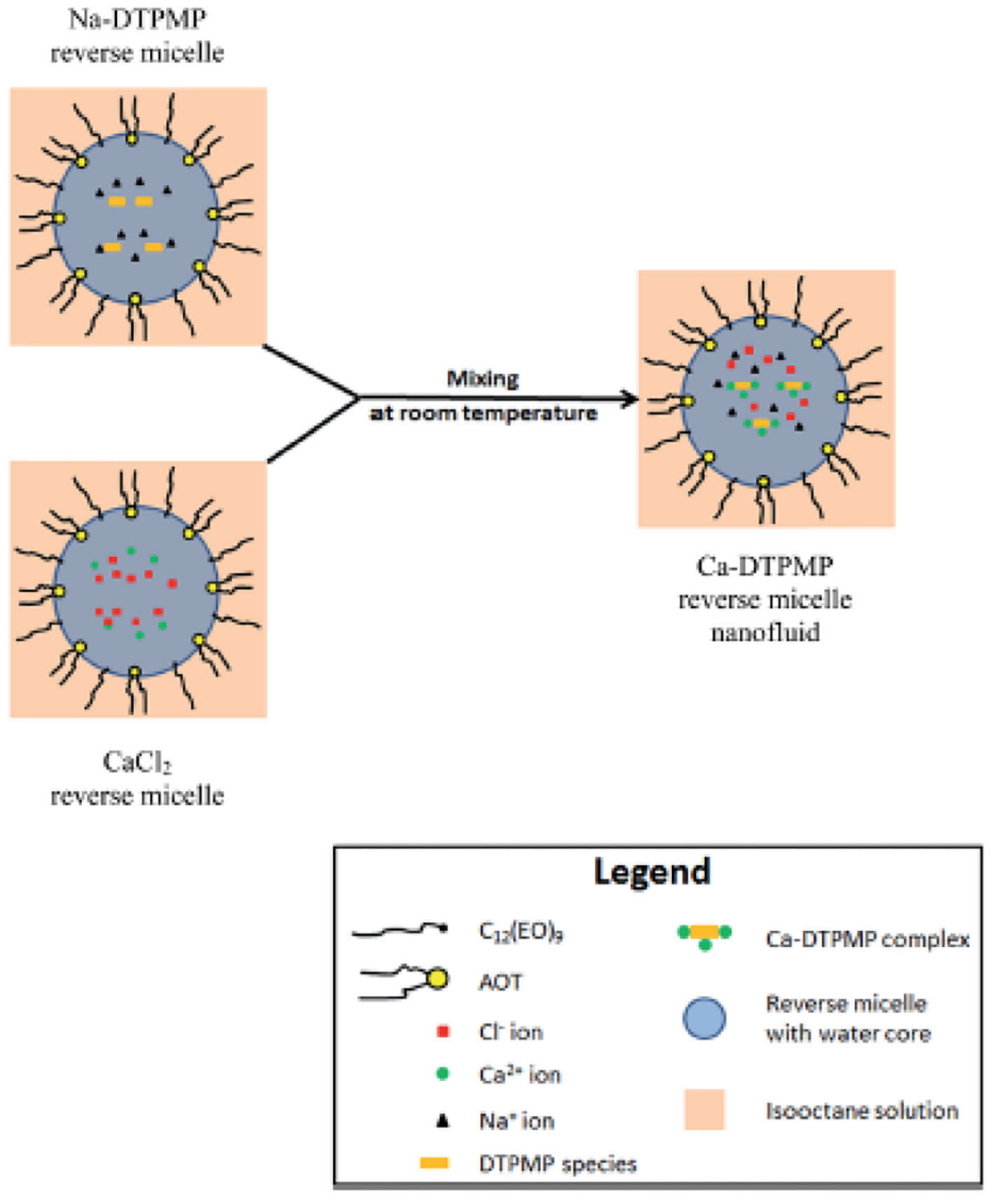
Schematic diagram of the synthesis of Ca-DTPMP reverse micelle nanofluid. Reproduced from Zhang et al. (2016d) with permission from The Royal Society of Chemistry.

Although the aforementioned Ca-DTPMP SINMs can significantly improve inhibitor performance in terms of transportability in formation medium and squeeze lifetime, these materials might not be suitable for application in the field characterized by low water cut and/or high water sensitivity (Collins and Hewartson, 2002; Guan et al., 2006; Vazquez et al., 2007, 2016; Chen et al., 2010). Since the aforementioned nanomaterials are water based, the water in the SINM package can damage the formation and impair well productivity due to mechanisms such as clay mobilization and pore constriction. Thus, it will be desirable to fabricate a non-aqueous SINM to be applied in low water-cut or water-sensitive wells for scale control. Therefore, a water-in-oil microemulsion (reverse micelle)-based Ca-DTPMP scale inhibitor nanomaterial fluid (nanofluid) was synthesis and tested at representative oilfield conditions (Zhang et al., 2016d). The synthesis of this reverse micelle nanofluid (RMNF) was achieved by mixing a calcium-containing microemulsion fluid with another DTPMP-based microemulsion solution at ambient condition with isooctane being the continuous phase, as illustrated in Figure 11. To facilitate RMNF preparation, a non-ionic and an anionic surfactant were adopted to reduce surface tension so as to increase the solubilization of the surfactant mixture in non-polar (isooctane) system. Upon mixing CaCl_2_ solution or DTPMP solution with surfactant mixture, a transparent solution can be obtained. The resultant Ca-DTPMP RMNF by mixing these two solutions contains over 90% organic (isooctane) domain with an average particle diameter of 250 nm. The molecular formula of the reverse micelle is Ca_2.8_H_4.4_DTPMP. The prepared Ca-DTPMP RMNF system was highly stable at 70°C and under centrifugal treatment.

**Figure 12:**
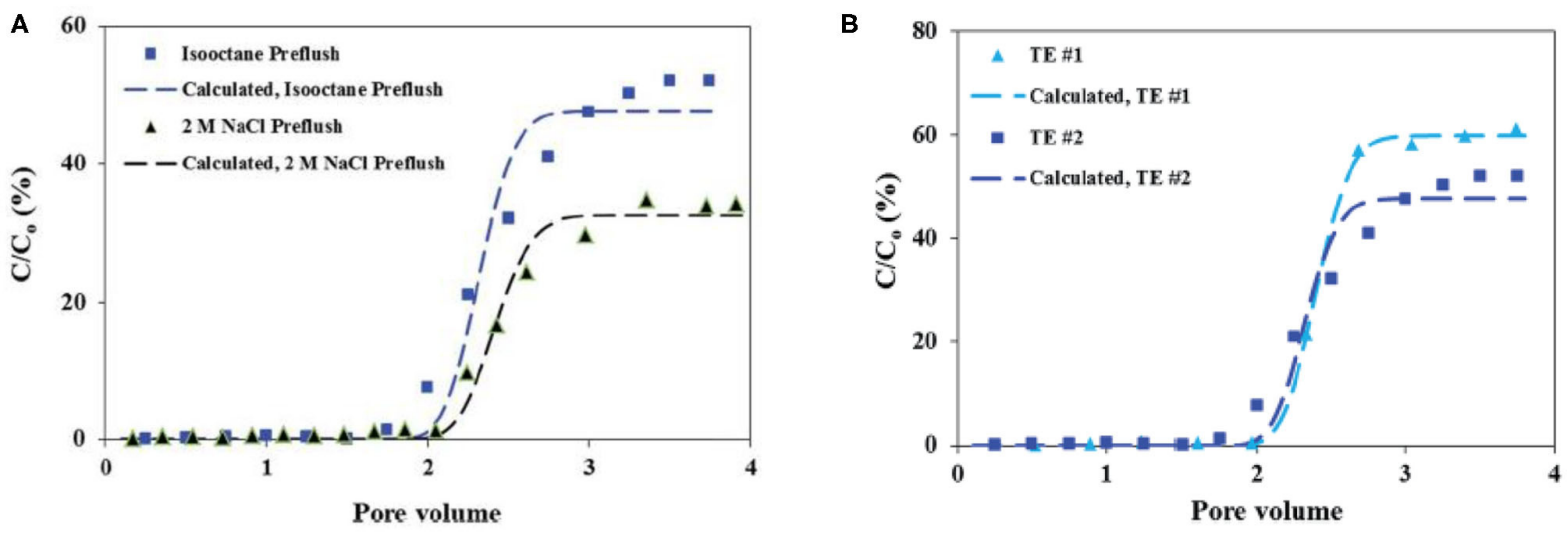
**(A)** Breakthrough profiles of inhibitor reverse micelle with preflush solution as isooctane or 2 M NaCl. Reproduced from Zhang et al. (2016d) with permission from The Royal Society of Chemistry. **(B)** Breakthrough curves of reverse micelle inhibitor nanomaterials at two different pore velocities of 5.73 and 2.85 cm min^−1^ in calcite medium with isooctane preflush (TE #1 and TE #2). Reproduced from Zhang et al. (2016e) with permission from The Royal Society of Chemistry. The markers represent the experimentally obtained breakthrough levels, while the dashed lines denote the calculated breakthrough levels based on the mathematical solution to Equation (1).

Transport experiments of the prepared Ca-DTPMP RMNF suggest that this material shows a reduced transportability in calcite medium, compared with the aforementioned aqueous Ca-DTPMP SINM surface modified by SDBS surfactant. The authors attributed the observation of the reduction in RMNF transportability to the weaker negative surface charge arising from anionic surfactant relative to SDBS. Furthermore, the impact of preflush on RMNF transport was evaluated experimentally. As elaborated above, preflush is a step prior to inhibitor pill injection in field squeeze treatment to clean up reservoir matrix. It was observed that preflushing the calcite medium with isooctane solution can significantly improve RMNF transportability relative to the experiments using 2 M NaCl as the preflush fluid, as shown in Figure 12A. The reason is that isooctane preflushing can remove the aqueous brine in formation pore space, coating the calcite particle with isooctane layer. On the other hand, 2 M NaCl solution preflushing will fill the pore space with aqueous brine, which can considerably reduce micellar system stability upon contact with RMNF. Furthermore, through another set of transport studies of RMNF, transport experimental results indicate that flowrate (pore velocity) can substantially influence the migration capacity of RMNF and breakthrough level can be improved by simply increasing the flowrate in porous medium (Zhang et al., 2016e). Enhanced transportability as a result of isooctane preflushing occurs at various flowrates, as shown in Figure 12B.

**Figure 13:**
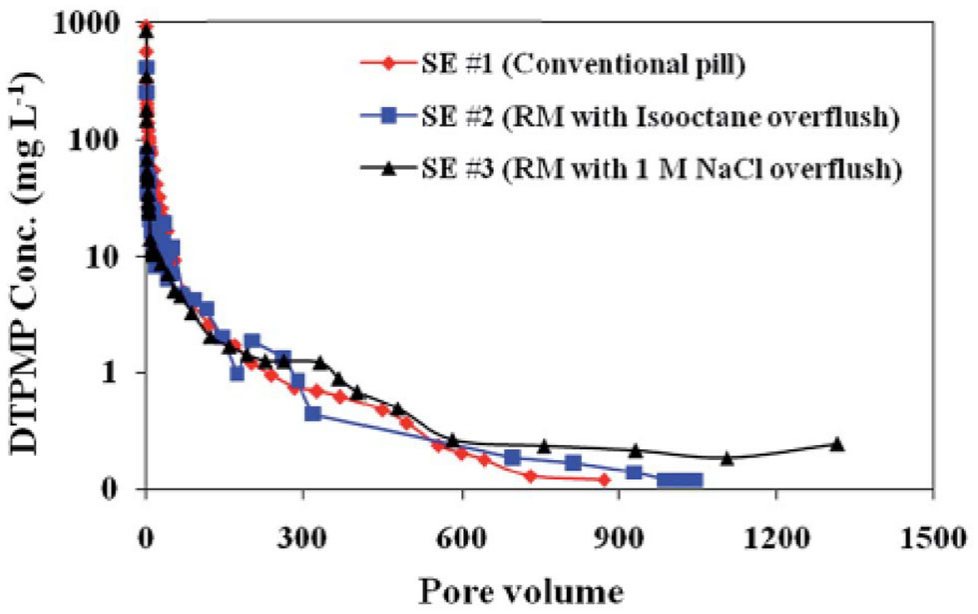
Squeeze simulation tests using reverse micelle inhibitor nanomaterials in calcite medium. Three DTPMP return profiles were included: SE #1 using conventional DTPMP pill; SE #2 using reverse micelle nanofluid with isooctane overflush; SE #3 using reverse micelle nanofluid with 1 M NaCl overflush. Reproduced from Zhang et al. (2016e) with permission from The Royal Society of Chemistry.

The long-term inhibitor return behavior of the prepared Ca-DTPMP RMNF was evaluated via a series of laboratory squeeze simulation tests. It shows that similar to the aforementioned aqueous SINM, the non-aqueous RMNF demonstrated a similar inhibitor return behavior with an extended squeeze lifetime (Zhang et al., 2016d,e). Particularly, experimental efforts were made to investigate the impact of different overflush fluids on inhibitor return. Overflush, as explained above, is a treatment step designated right after the completion of inhibitor pill injection in a field squeeze treatment to displace inhibitors into deeper formation zone. Laboratory investigations show that overflushing the injected RMNF with an aqueous brine (1 M NaCl) can noticeably enhance the return performance with an extended squeeze lifetime, relative to organic (isooctane) fluid overflushing, as depicted in Figure 13. This phenomenon can be explained by inhibitor adsorption–desorption dynamics and the difference in overflushing fluid in displacing inhibitor (Jordan et al., 2008b; Vazquez et al., 2009). Basically, aqueous fluid can effectively desorb the previously attached inhibitor from formation pore space and subsequently push the desorbed inhibitor into the second half of the column, leading to readsorption and reattachment of the inhibitor on the formation medium surface. This can allow more Ca-DTPMP RMNF to be retained by calcite surfaces, compared with isooctane overflushing.

### Ca-DTPMP Inhibitor Nanoparticles With Remaining Synthesis Fluid

In a recent study (Franco-Aguirre et al., 2018), experimental investigation was carried out to study the interaction between Ca-DTPMP and remaining synthesis fluid (RSF) for the objective of simultaneously inhibiting and removing the scale damage in tight gas–condensate reservoir formation. Synthesis of Ca-DTPMP SINM was realized with the aid of n-hexadecyl-trimethyl ammonium bromide (CTAB) surfactant. The supernatant fluid acquired from the filtration step is the RSF, which accounts for 30–40% of the initial volume. The particle size of the obtained SINM was between 36 and 69 nm. The authors reported that the combined effect of SINM and the RSF is satisfactory in inhibiting and removing scale-induced formation damage under dynamic conditions. The injection of the nanofluid can result in an increase in oil permeability, indicating that the nanofluid can inhibit scale formation, remediate formation damage, and stimulate hydrocarbon production. Moreover, the inhibition tests show a high perdurability of the nanofluid of around 60 PV prior to formation damage. The remediation tests suggest that the nanofluid treatment can recover formation system properties and improve the mobility of hydrocarbons.

### Ca-DTPMP Inhibitor Nanoparticles for Bulk Water Process Calcite Scaling Control

During water desalination process, mineral scale formation can also cause considerable damage to the processing facilities. An experimental study was reported to prepare Ca-DTPMP SINM to inhibit calcite scale to bulk water process (Kiaei and Haghtalab, 2014). The synthesis process was similar to the previous studies (Zhang et al., 2010, 2011a,b) where the surfactant CTAB was employed to regulate particle size. The authors adopted electrochemical measurement with regard to Ca^2+^ concentration and pH to obtain the precipitation/inhibition curves. An ion meter device was employed to study calcite precipitation kinetics. Experimental results suggest that SINM can effectively delay calcite precipitation kinetics and increase the final Ca^2+^ concentration in bulk solution. This observation was attributed to the interference of SINM with the crystal structure of calcite particles by considerably modifying the shape and morphology. The comparison of SINM with micro-Ca-DTPMP as well as commercial DTPMP product suggests that the SINM has an improved inhibition efficiency.

## Other Scale Inhibitor Nanomaterials

### Scale Inhibitor Nanoparticle Capsule

**Figure 14:**
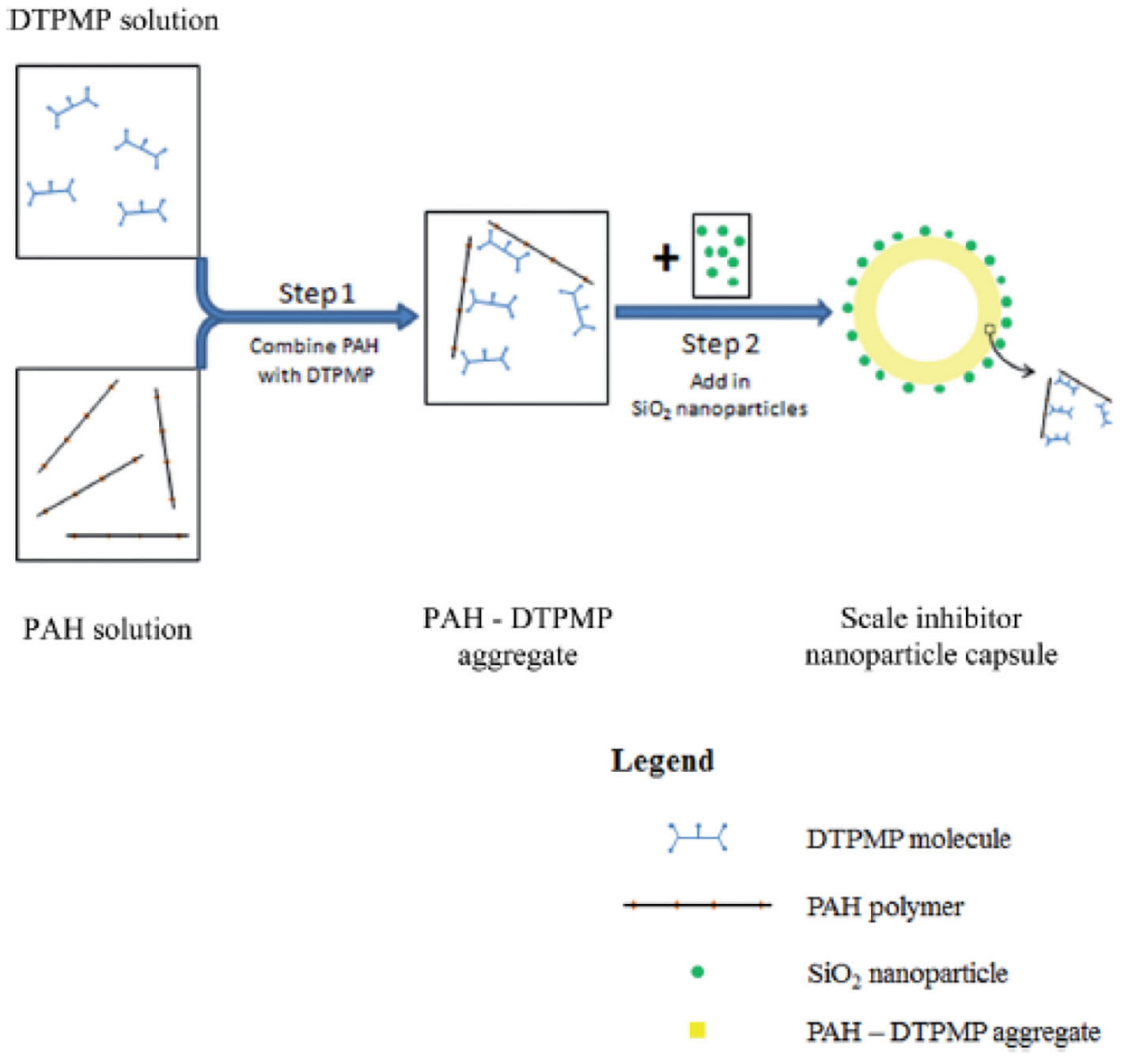
Schematic diagram of the synthesis of scale inhibitor nanoparticle capsule by mixing PAH and DTPMP solutions followed by addition of SiO_2_ nanoparticles. Reproduced from Zhang et al. (2016f) with permission from The Royal Society of Chemistry.

**Figure 15:**
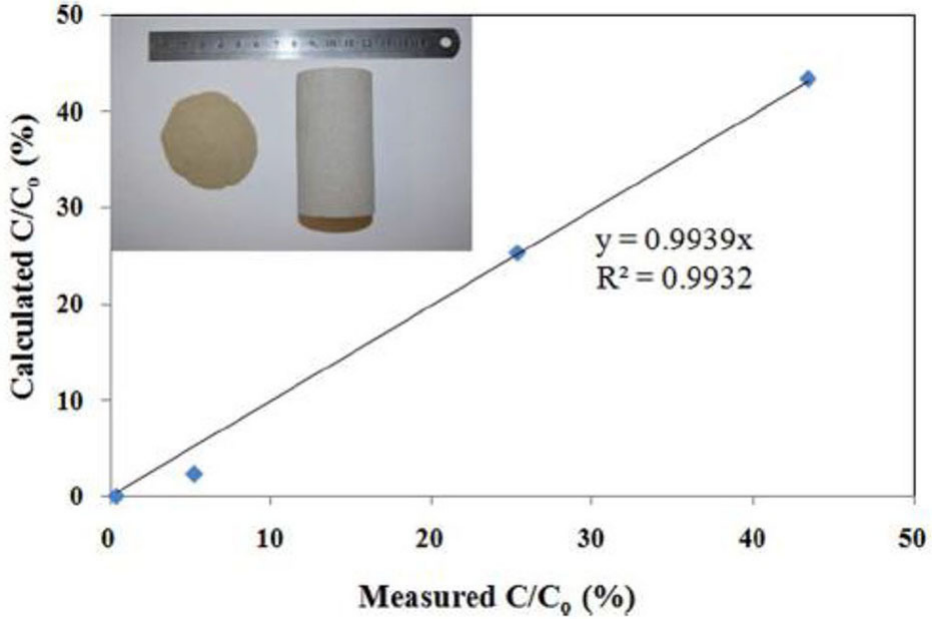
Breakthrough levels of scale inhibitor nanoparticle capsules in the consolidated sandstone core. The abscissas values are the experimentally obtained breakthrough levels; the ordinate values are calculated breakthrough levels from Equation (1). The insert is a photo of crushed sandstone grains with a grain size distribution of 0–106 μm on the left and a consolidated sandstone core on the right. Reproduced from Zhang et al. (2016f) with permission from The Royal Society of Chemistry.

Scale inhibitor nanoparticle capsule (SINC) was prepared in a laboratory setting to address the issue of transporting in consolidated formation core material (Zhang et al., 2016f). The previously introduced SINMs and RMNF are transportable in crushed formation materials including calcite and sandstone (Zhang et al., 2016b,c,d; Franco-Aguirre et al., 2018). As discussed by the authors, crushed formation materials can maintain chemical properties in terms of sorption and surface chemistry as those of the intact consolidated materials. However, crushed materials possess numerous micropores or cracks with a considerably higher pore throat size and elevated permeability. As a consequence, SINM can transport with a greatly reduced hindrance force in crushed formation medium relative to consolidated core. Thus, according to the authors, crushed formation materials are more appropriate to study onshore or shale field scale control where hydraulic fracturing treatment in the shale field can result in open cracks in the reservoir (Nash, 2010). On the other hand, it is more suitable to adopt intact consolidated formation materials for the sake of evaluating SINM migration in offshore deepwater field, which is commonly characterized by tight formation with a substantially lowered permeability and pore size. The synthesis of SINC was initiated by mixing DTPMP with a cationic polymer poly(allylamine hydrochloride) (PAH) to form PAH-DTPMP by interacting the phosphoryl oxygen of DTPMP with the amine group of PAH. Subsequently, silica (SiO_2_) nanoparticles were employed to form SiO_2_-PAH-DTPMP nanoparticle capsule with SiO_2_ as the building blocks. The synthesized SINC has a multilayered structure with the outside shell composed of SiO_2_ and inside core of PAH-DTPMP aggregate, as depicted in Figure 14. The migration capacity of SINC was initially evaluated by a column setup by adopting crushed formation materials. Experimental results show that the solution ionic strength of the preflush fluid, back pressure, and the system temperature can impact SINM breakthrough level in sandstone medium. More importantly, it shows that the prepared SINC can transport through a consolidated sandstone core, albeit a lower breakthrough level (Figure 15). This suggests that the synthesized SINC has the potential to migrate through tight formation.

### Cross-Link Scale Inhibitor Nanomaterials

As early as 2000, Bache and Nilsson (2000) presented a study of preparing ester scale inhibitors prepared by cross-linking various types of polycarboxylic acids for the goal of improving inhibitor retention and squeeze lifetime in the wellbore reservoir. The core flooding tests suggest that cross-linked inhibitor materials can considerably increase the treatment lifetime. Moreover, one can control the degree of product cross-linking by controlling esterification conditions and compositions. More recently, Yan et al. (2013) reported the synthesis and laboratory testing of aluminum (Al) cross-linked sulfonated polycarboxylic acid (Al-SPCA) inhibitor nanomaterial and microscale precipitates. The synthesis approach was based upon a urea-assisted hydrothermal procedure where Al^3+^ containing solution was mixed with another SPCA solution. Parameters such as Al^3+^ concentration, reaction time, and ionic strength were tested to control particle size. The particle size of the SINM can range from as low as 80 nm to several micrometers with a narrow size distribution. Furthermore, another common polymeric scale inhibitor PPCA was employed to mix with Al-SPCA solution to prepare PPCA-dispersed Al-SPCA SINM. Column transport experiments suggest that Al-SPCA without PPCA presence shows a reduced stability and migration capacity in the formation medium if the SINM is dispersed in 1% KCl solution. However, PPCA-dispersed Al-SPCA can reach almost 100% breakthrough in the same formation medium. Squeeze simulation tests show that the Al-SPCA SINM can extend squeeze lifetime beyond 3,800 PV. In another study, the same authors reported the synthesis of boehmite [**γ**-AlO(OH)] cross-linked polymeric SINM (Yan et al., 2014). Monodispersed boehmite nanoparticles were prepared to cross-link with SPCA to form AlO(OH)-SPCA SINM with an average particle size ranging from 20 to 60 nm with a narrow size distribution. This type of SINM can increase the SPCA inhibitor retention in formation medium by converting aqueous phase SPCA into a viscous gel. The authors carried out sorption studies to evaluate the attachment of SPCA onto Al(O)OH nanoparticles at different experimental conditions such as solution pH and aqueous Ca^2+^ concentration. It shows that solid phase SPCA can increase with a reduction in solution pH. Additionally, synthesis studies were carried out to prepare cross-linked SINM of Al(O)OH with PPCA and DTPMP. Squeeze simulation tests suggest that the presence of Ca^2+^ ion can improve the squeeze performance of SPCA cross-linked SINM to over 1,000 PV at or above 0.5 ppm.

### Metal-Oxide-Based Nanomaterials

Metal oxides in their own rights are not typically regarded as scale inhibitors. However, the presence of metal oxides can noticeably influence the functionality of scale inhibitor and/or affect mineral scale precipitation and deposition process. One type of metal oxide investigated for its role in scaling control is Al_2_O_3_ nanoparticle (NP). A recent study examined the role of the presence of Al_2_O_3_ nanofluid in calcite scale inhibition by the use of (2-phosphonobutane-1,2,4-tricarboxylic acid) (PBTCA) inhibitor (Wang et al., 2019). Laboratory studies suggest that the presence of Al_2_O_3_ nanoparticles can impair the scaling control performance of PBTCA inhibitor in the solution phase. It was found that Al_2_O_3_ NP substantially reduced calcite induction time and weakened PBTCA solubilization on calcite. On the other hand, surface scale inhibition experiments show that PBTCA performance was reduced at a lower Al_2_O_3_ NP concentration and improved at a higher Al_2_O_3_ NP concentration. It was believed that this phenomenon was related to sorption of Al_2_O_3_ NP to PBTCA and the modification of CaCO_3_ crystals due to the presence of NP. It was found that Al_2_O_3_ NP can distort CaCO_3_ crystal structure by promoting aragonite formation.

Another commonly investigated metal oxide for its impact in scaling control is silica (SiO_2_) NP (Safari et al., 2014). One study showed that the presence of silica NP can slow down solution conductivity reduction rate in an oversaturated solution, indicative of reducing scale deposition. The same group of authors also evaluated the impact of silica NP on DTPMP scale inhibitor performance in calcium sulfate scaling control (Golsefatan et al., 2016). Comparison was made among different scenarios of silica NP alone, DTPMP inhibitor alone, and silica NP/DTPMP blends. Experimental results showed that silica NP alone can reduce the rate of solution conductivity decrease where silica NP can serve as crystal growth inhibitor for calcite sulfate scale. On the other hand, the blends of silica NP with DTPMP inhibitor can substantially reduce solution solubility reduction rate, indicative of lowering calcium sulfate scale deposition, as long as the silica NP and inhibitor concentration can be optimized. It was clear that silica NP can considerably improve the effectiveness of scale control using DTPMP by reducing the mass of scale formed. More recently, these authors reported the experimental studies of silica on barite scale deposition kinetics during the process of water injection (Tavakoli et al., 2018). Factors such as temperature, mixing ratios, injection rate, and silica NP concentrations were evaluated for their roles in reducing the permeability of porous medium as a result of barite scale formation. It was reported that silica NP can prevent and delay the formation of barite scale during a water injection campaign. It was believed that the mechanism of silica NP in scaling control is related to the surface charge of the system. The presence of silica NP can substantially increase the surface charge, which can effectively suppress scale formation. Another study investigated the application of silica NP to control calcite sulfate scale at high temperature during water injection with high and low salinity (Moghadasi et al., 2019). It shows that the effectiveness of silica NP in controlling calcium sulfate scale is more pronounced at a higher salinity condition. However, the increase in system temperature can impair silica NP effectiveness. It was found that the impact of temperature on silica NP effectiveness is directly related to zeta potential of silica NP. In another study, experimental studies have been carried out to evaluate the feasibility to create a super hydrophobic surface with the aid of silica NP (Kumar et al., 2012). The said superhydrophobic surface is created on the surfaces of epoxy paint with a dip-coating process involving silica NP. The hydrophobicity of the surface can be further reinforced by another layer of aminopropyl polymer. Experimental studies show that this surface has a contact angle of 167.8°C with water and is highly stable in organic solvent. In another study, research work has been conducted to prepare magnetic iron oxide NP surface coated with maleic acid-2-acrylamido-2-methyl-1-propanesulfonate-based polymer (Do et al., 2013). Oleic acid coating on the surface of NP can enhance particle stability and activeness of NP in the polymerization stage. A core–shell structured composite NP with a particle size of 20–30 nm can be obtained. Scale inhibition tests suggest that this nanomaterial can effectively inhibit carbonate scale formation.

### Carbon-Based Nanomaterials

Similar to the investigation on metal oxide, experimental studies have been carried out to examine the impact of different carbon-based SINMs. In one study, experiment efforts have been made to examine the effect of carbon nanotubes (CNTs) on CaCO_3_ crystallization process via laboratory-scale inhibition test and instrumental analysis (Wan et al., 2019). It shows that CNT can effectively inhibit carbonate scale formation, which can be applicable in heat exchanger scale control. According to these authors, the role of CNT in scaling control is not through affecting CaCO_3_ solubility, which is confirmed via scaling ion concentration determination and solution conductivity measurement. Instead, CNT functions by modifying the crystalline structure of the deposited CaCO_3_ solid from calcite to aragonite, subjecting the scale particles to be difficult to be adsorbed on the surface of processing facilities. In another study (Ghorbani et al., 2012), laboratory studies have been conducted to investigate the adsorption of PPCA onto the surfaces of a carbon-based nanoparticle (CBNP) for the objective of improving the retention of PPCA inhibitor onto the surfaces of rock during a squeeze campaign. Competitive sorption study results show that PPCA molecules prefer to adsorb onto the surface of CBNP over various types of sands evaluated. This suggests that CBNP might have the potential to serve as an assistive mediator for PPCA inhibitor during a squeeze treatment. Table 1 summarizes a number of key aspects of the elaborated SINMs including synthesis approach, application, and key advantage.

**Table 1:** Summary of the scale inhibitor nanomaterials covered in this review.

**SINM type**	**Synthesis approach**	**Application**	**Key advantage**	**References**
Amorphous Ca-DTPMP scale inhibitor nanomaterials	Surfactant-assisted approach Silica-templated method	As the first nanomaterial of its kind, this SINM can migrate through formation materials and also release inhibitor during flow-back	Transportability in formation porous medium Return inhibitor during the long term flow-back	Zhang et al., 2010, 2011a
Crystalline Ca-DTPMP scale inhibitor nanomaterial	Diafiltration to develop into crystalline SINM. Citrate-assisted synthesis approach	Other than migration capacity, this material can return inhibitor with a significantly extended squeeze lifetime	Substantially extended squeeze lifetime can prolong squeeze lifetime	Zhang et al., 2011b, 2016c; Ruan et al., 2020
Reverse micelle Ca-DTPMP nanofluid	Mixing microemulsion fluids with surfactant presence	Applicable to fields characterized by low water cut and/or high water sensitivity	Applicability in low water cut fields or water sensitive wells	Zhang et al., 2016d,e
Ca-DTPMP inhibitor nanoparticles with remaining synthesis fluid	CTAB surfactant aided synthesis approach	Inhibiting and removal of scale-induced formation damage under dynamic conditions	Recover formation system properties and improve the mobility of hydrocarbons	Franco-Aguirre et al., 2018
Ca-DTPMP inhibitor nanoparticles for bulk water process calcite scaling control	CTAB surfactant adopted to regulate particle size	Effectively delay calcite precipitation kinetics and increase the final Ca^2+^ concentration in bulk solution	An improved inhibition efficiency for bulk water process calcite scaling control	Kiaei and Haghtalab, 2014
Scale inhibitor nanoparticle capsule	Combing cationic polymer with inhibitor with silica nanoparticle assistance	Transportability in consolidated formation material with a low permeability	Migration capacity through tight formation with a low permeability and porosity	Zhang et al., 2016f
Cross-link scale inhibitor nanomaterials	Urea-assisted hydrothermal procedure Cross-link boehmite nanoparticles with scale inhibitor	As an alternative method to prepare SINM, the prepared nanomaterials can transport information medium and return inhibitor	Transportability in formation porous medium Return inhibitor during the long-term flow-back	Yan et al., 2013, 2014
Metal oxide based nanomaterials	Depending on the type of metal oxide	Enhance inhibitor performance by increasing induction time; lowering scale deposition rate; and creating a super hydrophobic surface	The presence of metal oxide nanomaterials can improve scale inhibitor performance and distort scale crystal structure	Kumar et al., 2012; Do et al., 2013; Safari et al., 2014; Tavakoli et al., 2018; Moghadasi et al., 2019; Wang et al., 2019
Carbon based nanomaterials	Carbon based nanomaterials either purchased commercially or synthesized via established synthesis approach	Have the potential to serve as an assistive mediator for scale inhibitor during a squeeze treatment	These nanomaterials can modify scale crystal structure and can preferably adsorb scale inhibitor	Ghorbani et al., 2012; Wan et al., 2019

## Conclusions

Mineral scale deposition can lead to severe threats to the safety and integrity of oil and gas operations, particularly in shale and deepwater fields. To control scale threat, chemical inhibitors are widely deployed in both onshore and offshore operations. Although conventional inhibitors are effective in inhibiting scale threat, they have the drawbacks of short transport distance and limited squeeze lifetime due to their intrinsic chemical properties. This is especially true during squeeze treatment to control downhole and bottom well scale threats. In view of the fact that scale inhibitors form complex or precipitate with the metal ions in formation, the idea of fabricating a preprecipitated metal-inhibitor scale inhibitor nanomaterial (SINM) in a laboratory setting can be perceived. Extensive experimental studies have been carried out in the past decade to synthesize and evaluate various types of SINM for oilfield scale control. Based on the chemical compositions of SINMs, these functional nanomaterials can be generally categorized into metal-inhibitor SINM, nanoparticle capsule, cross-link scale inhibitor nanomaterials, metal-oxide-based nanomaterials, carbon-based nanomaterials, etc. Evaluation of these nanomaterials focuses on three areas including scale inhibition effectiveness, transportability in formation medium, and inhibitor return performance. As for inhibition effectiveness, depending on SINM chemical compositions, SINMs can either directly inhibit scale formation or influence chemical inhibitor performance. With regard to SINM migration, experimental results indicate that multiple factors can affect SINM migration in the formation porous medium, such as medium properties, SINM compositions, SINM surface chemistry, preflushing or overflush fluid, etc. In terms of inhibitor return performance, laboratory squeeze simulation tests suggest that amorphous metal-inhibitor SINMs exceed the return performance of conventional inhibitors. Furthermore, crystalline metal-inhibitor SINM obtained by development from amorphous materials demonstrated a significant extension of squeeze lifetime. Clearly, SINMs have the potential to be adopted in actual oilfield applications to control mineral scale threat. These materials can provide a promising alternative to conventional inhibitor chemicals with a superior transportability in formation media and an extended squeeze lifetime. However, additional experimental studies are needed to ensure a complete compatibility of these materials with formation materials at oilfield operating conditions. Ideally, transport and squeeze simulation tests can be conducted at representative oilfield conditions, i.e., high-temperature and high-pressure conditions with actual core materials. This involves additional laboratory efforts to investigate novel compositions and synthesis approaches of SINMs. The prepared SINMs need to be able to withstand the harsh environment in reservoir conditions with satisfactory transport behavior and prolonged squeeze lifetime. Based on the detailed elaboration in this review, it is clear that SINMs can expend our ability in controlling oilfield scale threat and have the potential to be applied in field scale control campaigns as an alternative engineering solution. The SINMs presented in this review exemplify the continuous development in our capabilities in adopting novel nanotechnology in combating actual engineering challenges in petroleum industry.

## Author Contributions

PZ conceived the concept of the review, drafted the manuscript, searched for updated bibliography, and prepared the figures.

## Conflict of Interest

The author declares that the research was conducted in the absence of any commercial or financial relationships that could be construed as a potential conflict of interest.

## Abbreviations

Al-SPCA: aluminum cross-linked sulfonated polycarboxylic acid
ATMP: aminotris(methylenephosphonic acid)
BHPMP: bis-hexamethylene triamine-penta(methylene phosphonic acid)
CBNP: carbon-based NP
CNT: carbon nanotubes
CTAB: n-hexadecyl-trimethyl ammonium bromide
DLVO: Derjaguin–Landau–Verwey–Overbeek
DTPMP: diethylenetriamine pentakis (methylene phosphonic acid)
MIC: minimum inhibitor concentration
NP: nanoparticle
PAA: polyacrylic acid
PAH: poly(allylamine hydrochloride)
PPCA: phosphino-polycarboxylic acid
PBTCA: (2-phosphonobutane-1,2,4-tricarboxylic acid)
PV: pore volume
RMNF: reverse micelle nanofluid
RSF: remaining synthesis fluid
SDBS: sodium dodecylbenzene sulfonate
SEM: scanning electron microscopy
SINC: scale inhibitor nanoparticle capsule
SINM: scale inhibitor nanomaterials
SPE: Society of Petroleum Engineers
XRD: X-ray diffraction.

## References

[B1] Bache Ø. Nilsson S. (2000). Ester cross-linking of polycarboxylic acid scale inhibitors as a possible means to increase inhibitor squeeze lifetime, in Presented at International Symposium on Oilfield Scale. SPE 60190 (Aberdeen).

[B2] Bera A. Belhaj H. (2016). Application of nanotechnology by means of nanoparticles and nanodispersions in oil recovery - a comprehensive review. J. Nat. Gas Sci. Eng. 34, 1284–1309. 10.1016/j.jngse.2016.08.023

[B3] Bukuaghangin O. Sanni O. Kapur N. Huggan M. Neville A. Charpentier T. (2016). Kinetics study of barium sulphate surface scaling and inhibition with a once-through flow system. J. Petrol. Sci. Eng. 147, 699–706. 10.1016/j.petrol.2016.09.035

[B4] Carvalho S. Palermo L. Boak L. Sorbie K. Lucas E. F. (2017). Influence of terpolymer based on amide, carboxylic, and sulfonic groups on the barium sulfate inhibition. Energy Fuels 31, 10648–10654. 10.1021/acs.energyfuels.7b01767

[B5] Chang H. Li T. Liu B. Vidic R. D. Elimelech M. Crittenden J. C. (2019). Potential and implemented membrane-based technologies for the treatment and reuse of flowback and produced water from shale gas and oil plays: a review. Desalination 455, 34–57. 10.1016/j.desal.2019.01.001

[B6] Charbeneau R. J. (2006). Groundwater Hydraulics and Pollutant Transport. Long Grove, IL: Waveland Press.

[B7] Chaussemier M. Pourmohtasham E. Gelus D. Pécoul N. Perrot H. Lédion J. (2015). State of art of natural inhibitors of calcium carbonate scaling. A review article. Desalination 356, 47–55. 10.1016/j.desal.2014.10.014

[B8] Chen P. Juliussen B. Vikane O. Montgomerie H. Benvie R. Froytlog C. (2010). Development of non-aqueous/low density scale inhibitor package for downhole squeeze treatments, in Presented at SPE International Conference on Oilfield Scale. SPE 130686 (Aberdeen).

[B9] Chen T. Neville A. Yuan M. (2005). Calcium carbonate scale formation–assessing the initial stages of precipitation and deposition. J. Petrol. Sci. Eng. 46, 185–194. 10.1016/j.petrol.2004.12.004

[B10] Clark M. M. (2009). Transport Modeling for Environmental Engineers and Scientists. 2nd ed Hoboken, NJ: John Wiley and Sons.

[B11] Collins I. R. Hewartson J. A. (2002). Extending squeeze lifetimes using miscible displacement, in Presented at SPE International Symposium on Oilfield Scale. SPE 74650 (Aberdeen).

[B12] Cosultchi A. Garciafigueroa E. Mar B. Garcia-Bórquez A. Lara V. H. Bosch P. (2002). Contribution of organic and mineral compounds to the formation of solid deposits inside petroleum wells. Fuel 81, 413–421. 10.1016/S0016-2361(01)00187-9

[B13] Cowan J. C. Weintritt D. J. (1976). Water-Formed Scale Deposits. Houston, TX: Gulf Publishing Company.

[B14] Dai Z. Zhang F. Kan A. T. Ruan G. Yan F. Bhandari N. (2019). Two-stage model reveals barite crystallization kinetics from solution turbidity. Indian Eng. Chem. Res. 58, 10864–10874. 10.1021/acs.iecr.9b01707

[B15] de Jonge L. W. Kjaergaard C. Moldrup P. (2004). Colloids and colloid-facilitated transport of contaminants in soils: an introduction. Vadose Zone J. 3, 321–325. 10.2136/vzj2004.0321

[B16] Do B. P. H. Nguyen B. D. Nguyen H. D. Nguyen P. T. (2013). Synthesis of magnetic composite nanoparticles enveloped in copolymers specified for scale inhibition application. Adv. Nat. Sci. Nanosci. Nanotechnol. 4:045016 10.1088/2043-6262/4/4/045016

[B17] Fan C. Kan A. Fu G. Tomson M. Shen D. (2010). Quantitative evaluation of calcium sulfate precipitation kinetics in the presence and absence of scale inhibitors. SPE J. 15, 977–988. 10.2118/121563-PA

[B18] Fan C. Kan A. Zhang P. Lu H. Work S. Yu J. (2012b). Scale prediction and inhibition for oil and gas production at high temperature/high pressure. SPE J. 17, 379–392. 10.2118/130690-PA

[B19] Fan C. Kan A. T. Zhang P. Tomson M. B. (2011). Barite nucleation and inhibition at 0-200°C, with and without hydrate inhibitors. SPE J. (2011). 16, 440–450. 10.2118/121559-PA

[B20] Fan C. Shi W. Zhang P. Lu H. Zhang N. Work S. (2012a). Ultrahigh-temperature/ultrahigh-pressure scale control for deepwater oil and gas production. SPE J. 17, 177–186. 10.2118/141349-PA

[B21] Farooqui N. M. Sorbie K. S. (2016). The use of PPCA in scale-inhibitor precipitation squeezes: solubility, inhibition efficiency, and molecular-weight effects. SPE Prod. Oper. 31, 258–269. 10.2118/169792-PA

[B22] Fink J. (2011). Petroleum Engineer's Guide to Oil Field Chemicals and Fluids. Waltham, MA: Gulf Professional Publishing.

[B23] Franco-Aguirre M. Zabala R. D. Lopera S. H. Franco C. A. Cortés F. B. (2018). Ca-DTPMP nanoparticles-based nanofluids for the inhibition and remediation of formation damage due to CaCO_3_ scaling in tight gas-condensate reservoirs. J. Petrol. Sci. Eng. 169, 636–645. 10.1016/j.petrol.2018.06.021

[B24] Frenier W. W. Ziauddin M. (2008). Formation, Removal, and Inhibition of Inorganic Scale in the Oilfield Environment. Richardson, TX: Society of Petroleum Engineers.

[B25] Gao S. House W. Chapman W. G. (2006). Detecting gas hydrate behavior in crude oil using NMR. J. Phys. Chem. B. 110, 6549–6552. 10.1021/jp055039a 16570953

[B26] Ghorbani N. Wilson M. Kapur N. Fleming N. Neville A. (2012). Using nanoscale dispersed particles to assist in the retention of polyphosphinocarboxylic acid (PPCA) scale inhibitor on rock, in Presented at SPE International Oilfield Nanotechnology Conference and Exhibition. SPE 156200 (Noordwijk).

[B27] Golsefatan A. R. Safari M. Jamialahmadi M. (2016). Using silica nanoparticles to improve DETPMP scale inhibitor performance as a novel calcium sulfate inhibitor. Desalinat. Water Treat. 57, 20800–20808. 10.1080/19443994.2015.1119742

[B28] Guan H. Sorbie K. S. Mackay E. J. (2006). The comparison of non-aqueous and aqueous scale inhibitor treatments: experimental and modeling studies. SPE Prod. Oper. 21, 419–429. 10.2118/87445-PA

[B29] Gudmundsson J. S. (2017). Flow Assurance Solids in Oil and Gas Production. Leidon: CRC Press.

[B30] Haindade Z. M. W. Bihani A. D. Javeri S. M. Jere C. B. (2012). Enhancing flow assurance using Co-Ni nanoparticles for dewaxing of production tubing, in Presented at SPE International Oilfield Nanotechnology Conference and Exhibition. SPE-157119 (Noordwijk).

[B31] Hajirezaie S. Wu X. Soltanian M. R. Sakhad S. (2019). Numerical simulation of mineral precipitation in hydrocarbon reservoirs and wellbores. Fuel 238, 462–472. 10.1016/j.fuel.2018.10.101

[B32] Hu Y. Mackay E. (2017). Modeling of geochemical reactions occurring in the Gyda field under cold-seawater injection on the basis of produced-water-chemistry data and implications for scale management. SPE Prod. Oper. 32, 449–468. 10.2118/179911-PA

[B33] Ishkov O. Guarnieri R. Jordan M. M. Mackay E. (2019). Impact of temperature on scale formation in chalk reservoirs. SPE Prod. Oper. 34, 332–343. 10.2118/185772-PA

[B34] Jarrahian K. Sorbie K. S. (2020). Mechanistic investigation of adsorption behavior of two scale inhibitors on carbonate formations for application in squeeze treatments. Energy Fuels 34, 4484–4496. 10.1021/acs.energyfuels.0c00326

[B35] Jordan M. M. Collins I. R. Mackay E. J. (2008a). Low sulfate seawater injection for barium sulfate scale control: a life-of-field solution to a complex challenge. SPE Prod. Oper. 23, 192–209. 10.2118/98096-PA

[B36] Jordan M. M. Mackay E. J. Vazquez O. (2008b). The influence of overflush fluid type on scale squeeze life time – field examples and placement simulation evaluation, in Presented at NACE International. NACE 08356 (New Orleans).

[B37] Kan A. Tomson M. (2012). Scale prediction for oil and gas production. SPE J. 17, 362–378. 10.2118/132237-PA

[B38] Kan A. T. Dai J. Z. Deng G. Harouaka K. Lu Y-T. Wang X. (2019). Recent advances in scale prediction: approach and limitations. SPE J. 24, 2209–2220. 10.2118/190754-PA

[B39] Kan A. T. Fu G. Tomson M. B. Al-Thubaiti M. Xiao A. J. (2004). Factors affecting scale inhibitor retention in carbonate-rich formation during squeeze treatment. SPE J. 9, 280–289. 10.2118/80230-PA

[B40] Kan A. T. Oddo J. E. Tomson M. B. (1994). Formation of two calcium diethylenetriaminepentakis(methylene phosphonic acid) precipitates and their physical chemical properties. Langmuir 10, 1450–1455. 10.1021/la00017a022

[B41] Kelland M. A. (2014). Production Chemicals for the Oil and Gas Industry. 2nd ed Boca Raton, FL: CRC Press.

[B42] Kelland M. A. (2006). History of the development of low dosage hydrate inhibitors. Energy Fuels 20, 825–847. 10.1021/ef050427x

[B43] Kelland M. A. (2011). Effect of various cations on the formation of calcium carbonate and barium sulfate scale with and without scale inhibitors. Ind. Eng. Chem. Res. 50, 5852–5861. 10.1021/ie2003494

[B44] Kiaei Z. Haghtalab A. (2014). Experimental study of using Ca-DTPMP nanoparticles in inhibition of CaCO_3_ scaling in a bulk water process. Desalination 338, 84–92. 10.1016/j.desal.2014.01.027

[B45] Kini G. C. Yu J. Wang L. Kan A. T. Biswal S. L. Tour J. M. (2014). Salt- and temperature-stable quantum dot nanoparticles for porousmedia flow. Colloids Surf. A. 443, 492–500. 10.1016/j.colsurfa.2013.11.042

[B46] Kong X. Ohadi M. (2010). Application of micro and nano technologies in oil and gas industry - an overview of the recent progress, in Presented at Abu Dhabi International Petroleum Exhibition and Conference. SPE 138241 (Abu Dhabi).

[B47] Kumar D. Chishti S. S. Rai A. Patwardhan S. D. (2012). Scale inhibition using nano-silica particles, in Presented at SPE Middle East Health, Safety, Security, and Environment Conference and Exhibition. SPE 149321 (Abu Dhabi).

[B48] Letchford K. Burt H. (2007). A review of the formation and classification of amphiphilic block copolymer nanoparticulate structures: micelles, nanospheres, nanocapsules and polymersomes. Eur. J. Pharm. Biopharm. 65, 259–269. 10.1016/j.ejpb.2006.11.009 17196803

[B49] Li Z. Linares R. V. Bucs S. Aubry C. Ghaffour N. Vrouwenvelder J. S. (2015). Calcium carbonate scaling in seawater desalination by ammonia–carbon dioxide forward osmosis: mechanism and implications. J. Membrane Sci. 481, 36–43. 10.1016/j.memsci.2014.12.055

[B50] Liu H. Jin X. Ding B. (2016). Application of nanotechnology in petroleum exploration and development. Pet. Explor. Dev. 43, 1107–1115. 10.1016/S1876-3804(16)30129-X

[B51] Liu Y. Kan A. Zhang Z. Yan C. Yan F. Zhang F. (2016). An assay method to determine mineral scale inhibitor efficiency in produced water. J. Petrol. Sci. Eng. 143, 103–112. 10.1016/j.petrol.2016.02.024

[B52] Mackay E. (2003). Predicting in situ sulphate scale deposition and the impact on produced ion concentrations. Chem. Eng. Res. Des. 81, 326–332. 10.1205/02638760360596874

[B53] Mackay E. J. Jordan M. M. Feasey N. D. Shah D. J. Kumar P. S. Ali S. A. (2005). Integrated risk analysis for scale management in deepwater developments. SPE Prod. Fac. 20, 138–154. 10.2118/87459-PA

[B54] Moghadasi R. Rostami A. Tatar A. Hemmati-Sarapardeh A. (2019). An experimental study of Nanosilica application in reducing calcium sulfate scale at high temperatures during high and low salinity water injection. J. Pet. Sci. Eng. 179, 7–18. 10.1016/j.petrol.2019.04.021

[B55] Mohammadi M. Akbari M. Fakhroueian Z. Bahramian A. Azin R. Arya S. (2011). Inhibition of asphaltene precipitation by TiO_2_, SiO_2_, and ZrO_2_ nanofluids. Energy Fuels 25, 3150–3156. 10.1021/ef2001635

[B56] Nash K. M. (2010). Shale Gas Development. Hauppauge, NY: Nova Science Publishers, Inc.

[B57] Ness G. Sorbie K. S. (2019). Rigorous carbonate and sulfide scale predictions: what really matters? Energy Fuels 33, 10765–10774. 10.1021/acs.energyfuels.9b02646

[B58] Olayiwola S. O. Dejam M. (2019). A comprehensive review on interaction of nanoparticles with low salinity water and surfactant for enhanced oil recovery in sandstone and carbonate reservoirs. Fuel 241, 1045–1057. 10.1016/j.fuel.2018.12.122

[B59] Qasim A. Khan M. S. Lal B. Shariff A. M. (2019). A perspective on dual purpose gas hydrate and corrosion inhibitors for flow assurance. J. Petrol. Sci. Eng. 183:106418 10.1016/j.petrol.2019.106418

[B60] Ribeiro A. S. Silva D. Mackay E. J. Sorbie K. (2017). The impact of vapor/liquid-equilibria calculations on scale-prediction modeling. SPE Prod. Oper. 3 2, 64–72. 10.2118/179885-PA

[B61] Ruan G. Kan A. T. Tomson M. B. Zhang P. (2020). Facile one-pot synthesis of metal-phosphonate colloidal scale inhibitor: synthesis and laboratory evaluation. Fuel 282:118855 10.1016/j.fuel.2020.118855

[B62] Ryan J. N. Elimelech M. (1996). Colloid mobilization and transport in groundwater, Colloids Surf. A. 107, 1–56. 10.1016/0927-7757(95)03384-X

[B63] Safari M. Golsefatan A. Jamialahmadi M. (2014). Inhibition of scale formation using silica nanoparticle. J. Dispers. Sci. Technol. 35, 1502–1510. 10.1080/01932691.2013.840242

[B64] Sanni O. Bukuaghangin O. Huggan M. Kapur N. Charpentier T. Neville A. (2017). Development of a novel once-through flow visualization technique for kinetic study of bulk and surface scaling. Rev. Sci. Instrum. 88:103903. 10.1063/1.4991729 29092516

[B65] Setta F.-A. Neville A. (2011). Efficiency assessment of inhibitors on CaCO_3_ precipitation kinetics in the bulk and deposition on a stainless steel surface (316 L). Desalination 281, 340–347. 10.1016/j.desal.2011.08.021

[B66] Shaw S. S. Sorbie K. Boak L. S. (2012a). The effects of barium sulfate saturation ratio, calcium, and magnesium on the inhibition efficiency - Part I: phosphonate scale inhibitors. SPE Prod. Oper. 27, 306–317. 10.2118/130373-PA

[B67] Shaw S. S. Sorbie K. Boak L. S. (2012b). The effects of barium sulfate saturation ratio, calcium, and magnesium on the inhibition efficiency: Part II: polymeric scale inhibitors. SPE Prod. Oper. 27, 390–403. 10.2118/130374-PA

[B68] Shaw S. S. Sorbie K. S. (2014). Structure, stoichiometry, and modeling of calcium phosphonate scale-inhibitor complexes for application in precipitation-squeeze processes. SPE Prod. Oper. 29, 139–151. 10.2118/164051-PA

[B69] Shen D. Zhang P. Kan A. T. Fu G. Alsaiari H. A. Tomson M. B. (2008). Control placement of scale inhibitors in the formation with stable Ca-DTPMP nanoparticle suspension and its transport in porous medium, in Presented at SPE International Oilfield Scale Conference (Aberdeen).

[B70] Shi W. Kan A. T. Zhang N. Tomson M. (2013). Dissolution of calcite at up to 250 C and 1450 bar and the presence of mixed salts. Ind. Eng. Chem. Res. 52, 2439–2448. 10.1021/ie302190e

[B71] Sorbie K. S. Wesselingh E. M. Yuan M. D. Lemanczyk R. Z. Todd A. C. (1997). Scale inhibitor squeeze strategies in horizontal wells. J. Can. Petrol. Technol. 36, 27–35. 10.2118/97-05-01

[B72] Stamatiou A. Sorbie K. S. (2020). Analytical solutions for a 1D scale inhibitor transport model with coupled adsorption and precipitation. Transp. Porous Media 132, 591–625. 10.1007/s11242-020-01405-0

[B73] Tavakoli H. M. Jamialahmadi M. Kord S. Daryasafar A. (2018). Experimental investigation of the effect of silica nanoparticles on the kinetics of barium sulfate scaling during water injection process. J. Petrol. Sci. Eng. 169, 344–352. 10.1016/j.petrol.2018.05.077

[B74] Tomson M.B. Kan A.T. Fu G. Shen D. Nasr-El-Din H.A. Al-Saiari H. (2008). Mechanistic understanding of rock/phosphonate interactions and the effect of metal ions on inhibitor retention. SPE J. 13, 325–336. 10.2118/100494-PA

[B75] Tomson M. B. Kan A. T. Fu G. (2006). Control of inhibitor squeeze via mechanistic understanding of inhibitor chemistry. SPE J. 11, 283–293. 10.2118/87450-PA

[B76] Tomson M. B. Kan A. T. Oddo J. E. (1994). Acid/Base and metal complex solution chemistry of the polyphosphonate DTPMP versus temperature and ionic strength. Langmuir 10, 1442–1449. 10.1021/la00017a021

[B77] Touati K. Alia E. Zendah H. Elfil H. Hannachi A. (2018). Sand filters scaling by calcium carbonate precipitation during groundwater reverse osmosis. Desalination 430, 24–32. 10.1016/j.desal.2017.12.037

[B78] Vankeuren A. N. P. Hakala J. A. Jarvis K. Moore J. E. (2017). Mineral reactions in shale gas reservoirs: barite scale formation from reusing produced water as hydraulic fracturing fluid. Environ. Sci. Technol. 51, 9391–9402. 10.1021/acs.est.7b01979 28723084

[B79] Vazirian M. M. Charpentier T. V. J. Oliveira Penna M. Neville A. (2016). Surface inorganic scale formation in oil and gas industry: as adhesion and deposition processes. J. Petrol. Sci. Eng. 137, 22–32. 10.1016/j.petrol.2015.11.005

[B80] Vazquez O. Corne D. Mackay E. Jordan M. M. (2013). Automatic isotherm derivation from field data for oilfield scale-inhibitor squeeze treatments. SPE J. 18, 563–574. 10.2118/154954-PA

[B81] Vazquez O. Herrero P. Mackay E. Jordan M. (2016). Non-aqueous vs aqueous overflush scale inhibitor squeeze treatment in an oilfield offshore Norway. J. Petrol. Sci. Eng. 138, 1–10. 10.1016/j.petrol.2015.11.033

[B82] Vazquez O. Mackay E. Tjomsland T. Nygård O. K. Storås E. (2014). Use of tracers to evaluate and optimize scale-squeeze-treatment design in the Norne field. SPE Prod. Oper. 29, 5–13. 10.2118/164114-PA

[B83] Vazquez O. Mackay E. J. Jordan M. M. (2009). Modeling the impact of diesel vs. water overflush fluids on scale-squeeze-treatment lives using a two-phase near-wellbore simulator. SPE Prod. Oper. 24, 473–480. 10.2118/114105-PA

[B84] Vazquez O. Mackay E. J. Sorbie K. S. (2007). Modelling of non-aqueous and aqueous scale inhibitor squeeze treatments, Presented at SPE International Symposium on Oilfield Chemistry (Houston, Texas). 10.2118/106422-MS

[B85] Vazquez O. Ross G. Jordan M. M. Baskoro D. A. A. Mackay E. Johnston C. (2019). Automatic optimization of oilfield-scale-inhibitor squeeze treatments delivered by diving-support vessel. SPE J. 24, 60–70. 10.2118/184535-PA

[B86] Vazquez O. Thanasutives P. Eliasson C. Fleming N. Mackay E. (2011). Modelling the application of scale-inhibitor-squeeze retention-enhancing additives. SPE Prod. Oper. 26, 270–277. 10.2118/141384-MS

[B87] Wan C. Wang L.-T. Sha J.-Y. Ge H.-H. (2019). Effect of carbon nanoparticles on the crystallization of calcium carbonate in aqueous solution. Nanomaterials 9:179. 10.3390/nano9020179 30717114 PMC6409635

[B88] Wang L.-T. Ge H.-H. Han Y.-T. Wan C. Sha J.-Y. Sheng K. (2019). Effects of Al_2_O_3_ nanoparticles on the formation of inorganic scale on heat exchange surface with and without scale inhibitor. Appl. Therm. Eng. 151, 1–10. 10.1016/j.applthermaleng.2019.01.075

[B89] Wang W. Kan A. Tomson M. (2013). A novel and comprehensive study of polymeric and traditional phosphonate inhibitors for high-temperature scale control. SPE J. 18, 575–582. 10.2118/155108-PA

[B90] Wang W. Kan A. T. Zhang F. Yan C. Tomson M. (2014). Measurement and prediction of thermal degradation of scale inhibitors. SPE J. 19, 1169–1176. 10.2118/164047-PA

[B91] Wang Z. Zhao Y. Zhang J. Pan S. Yu J. Sun B. (2018). Flow assurance during deepwater gas well testing: Hydrate blockage prediction and prevention. J. Petrol. Sci. Eng. 163, 211–216. 10.1016/j.petrol.2017.12.093

[B92] Webster N. A. S. Gan B. K. Livk I. (2014). *In situ* X-ray diffraction analysis of the onset of mineral scale deposition from synthetic oilfield processing waters. Fuel 137, 211–215. 10.1016/j.fuel.2014.07.089

[B93] Wheaton R. (2016). Fundamentals of Applied Reservoir Engineering: Appraisal, Economics and Optimization. Cambridge, MA: Gulf Professional Publishing.

[B94] Wu J. He J. Torsater O. Zhang Z. (2012). Effect of nanoparticles on oil-water flow in a confined nanochannel: a molecular dynamics Study, in Presented at SPE International Oilfield Nanotechnology Conference and Exhibition (Noordwijk).

[B95] Yan C. Kan A. Wang W. Wang L. Tomson M. (2013). Synthesis and size control of monodispersed Al-sulfonated polycarboxylic acid nanoparticles and their transport in porous media. SPE J. 18, 610–619. 10.2118/155627-PA

[B96] Yan C. Kan A. T. Wang W. Yan F. Wang L. Tomson M. (2014). Sorption study of γ-AlO(OH) nanoparticle-crosslinked polymeric scale inhibitors and their improved squeeze performance in porous media. SPE J. 19, 687–694. 10.2118/164086-PA

[B97] Yan C. Kan A. T. Zhang F. Liu Y. Tomson R. C. Tomson M. B. (2015). Systematic study of barite nucleation and inhibition with various polymeric scale inhibitors by novel laser apparatus. SPE J. 20, 642–651. 10.2118/169787-PA

[B98] Yan F. Dai Z. Ruan G. Alsaiari H. Bhandari N. Zhang F. (2017). Barite scale formation and inhibition in laminar and turbulent flow: a rotating cylinder approach. J. Petrol. Sci. Eng. 149, 183–192. 10.1016/j.petrol.2016.10.030

[B99] Yan F. Zhang F. Bhandari N. Wang L. Dai Z. Zhang Z. (2015). Adsorption and precipitation of scale inhibitors on shale formations. J. Petrol. Sci. Eng. 136, 32–40. 10.1016/j.petrol.2015.11.001

[B100] Yang J. Ji S. Li R. Qin W. Lu Y. (2015). Advances of nanotechnologies in oil and gas industries. Energy Explore Exploit. 33, 639–657. 10.1260/0144-5987.33.5.639

[B101] Zerpa L. E. Salager J.-S. Koh C. A. Sloan E. D. Sum A. K. (2011). Surface chemistry and gas hydrates in flow assurance. Ind. Eng. Chem. Res. 50, 188–197. 10.1021/ie100873k

[B102] Zhang P. Fan C. Lu H. Kan A. T. Tomson M. B. (2011a). Silica-templated synthesis of zinc-DTPMP nanoparticles, their transport in carbonate and sandstone porous media and scale inhibition. SPE J. 16, 662–671. 10.2118/130639-PA

[B103] Zhang P. Fan C. Lu H. Kan A. T. Tomson M. B. (2011b). Synthesis of crystalline phase silica-based Ca- DTPMP nanomaterials and their transport in carbonate and sandstone porous media. Ind. Eng. Chem. Res. 50, 1819–1830. 10.1021/ie101439x

[B104] Zhang P. Harris L. Demiroglu M. Gokool A. (2017a). Production chemistry: Exposing hidden treasures or generating complications? in Presented at Proceeding of Offshore Technology Conference (Houston, TX).

[B105] Zhang P. Kan A. T. Tomson M. B. (2016c). Enhanced transport of novel crystalline phase calcium-phosphonate nanomaterials and their long term flow back performance in laboratory squeeze simulation tests. RSC Adv. 6, 5259–5269. 10.1039/C5RA19618C

[B106] Zhang P. Liu Y. Kan A. T. Tomson M. B. (2019c). Laboratory evaluation of synergistic effect of transition metals with mineral scale inhibitor in controlling halite scale deposition. J. Petrol. Sci. Eng. 175, 120–128. 10.1016/j.petrol.2018.12.036

[B107] Zhang P. Liu Y. Kuok S. C. Kan A. T. Tomson M. B. (2019b). Development of modeling approaches to describe mineral scale deposition kinetics in porous medium and pipe flow system. J. Petrol. Sci. Eng. 178, 594–601. 10.1016/j.petrol.2019.03.070

[B108] Zhang P. Liu Y. Zhang N. Ip W. F. Kan A. T. Tomson M. B. (2019d). A novel attach-and-release mineral scale control strategy: laboratory investigation of retention and release of scale inhibitor on pipe surface. J. Ind. Eng. Chem. 70, 462–471. 10.1016/j.jiec.2018.11.009

[B109] Zhang P. Ruan G. Shen D. Kan A. T. Tomson M. B. (2016e). Transport and return of scale inhibitor reverse micelle nanofluid: impact of preflush and overflush. RSC Adv. 6, 66672–66681. 10.1039/C6RA07445F

[B110] Zhang P. Ruan G. Kan A. T. Tomson M. B. (2016f). Functional scale inhibitor nanoparticle capsule delivery vehicles for oilfield mineral scale control. RSC Adv. 6, 43016–43027. 10.1039/C6RA05427G

[B111] Zhang P. Shen D. Fan C. Kan A. T. Tomson M. B. (2010). Surfactant-assisted synthesis of metal- phosphonate nanoparticles and their transport in porous media. SPE J. 15, 610–617. 10.2118/121552-PA

[B112] Zhang P. Shen D. Kan A. T. Tomson M. B. (2016a). Phosphino-polycarboxylic acid modified scale inhibitor nanomaterial for oilfield scale control: transport and inhibitor return in formation media. RSC Adv. 6, 59195–59205. 10.1039/C6RA09973D

[B113] Zhang P. Shen D. Kan A. T. Tomson M. B. (2016d). Synthesis of novel calcium-phosphonate reserve micelle nanofluid for oilfield mineral scale control. RSC Adv. 6, 39883–39895. 10.1039/C6RA01228K

[B114] Zhang P. Shen D. Ruan G. Kan A. T. Tomson M. B. (2016b). Mechanistic understanding of calcium–phosphonate solid dissolution and scale inhibitor return behavior in oilfield reservoir: formation of middle phase. Phys. Chem. Chem. Phys. 18, 21458–21468. 10.1039/C6CP03148J 27426410

[B115] Zhang P. Shen D. Ruan G. Kan A. T. Tomson M. B. (2017b). Phosphino-polycarboxylic acid modified scale inhibitor nanomaterial: synthesis, characterization and migration. J. Ind. Engr. Chem. 45, 366–374. 10.1016/j.jiec.2016.10.004

[B116] Zhang P. Zhang N. Liu Y. Lu Y. T. Kan A. T. Tomson M. B. (2018). Application of a novel tube reactor for investigation of calcium carbonate mineral scale deposition kinetics. Chem. Eng. Res. Des. 137, 113–124. 10.1016/j.cherd.2018.07.001

[B117] Zhang P. Zhang Z. Zhu J. Kan A. T. Tomson M. B. (2019a). Experimental evaluation of common sulfate mineral scale coprecipitation kinetics in oilfield operating conditions. Energy Fuels 33, 6177–6186. 10.1021/acs.energyfuels.9b01030

[B118] Zhang T. Murphy M. J. Yu H. Bagaria H. G. Yoon K. Y. Nielson B. M. (2015). Investigation of nanoparticle adsorption during transport in porous media. SPE J. 20, 667–677. 10.2118/166346-PA

[B119] Zhang Z. Zhang P. Li Z. Kan A. T. Tomson M. B. (2018). Laboratory evaluationand mechanistic understanding of the impact of ferric species on oilfield scale inhibitor performance. Energy Fuels 32, 8348–8357. 10.1021/acs.energyfuels.8b01837

[B120] Zhang P. Kan A. T. Tomson M. B. (2015). Oil field mineral scale control, in Mineral Scales and Deposits: Scientific and Technological Approaches, eds Amjad Z. Demadis K. (Amsterdam: Elsevier Publishing), 603–617.

